# A lineage-specific Exo70 is required for receptor kinase–mediated immunity in barley

**DOI:** 10.1126/sciadv.abn7258

**Published:** 2022-07-06

**Authors:** Samuel Holden, Molly Bergum, Phon Green, Jan Bettgenhaeuser, Inmaculada Hernández-Pinzón, Anupriya Thind, Shaun Clare, James M. Russell, Amelia Hubbard, Jodi Taylor, Matthew Smoker, Matthew Gardiner, Laura Civolani, Francesco Cosenza, Serena Rosignoli, Roxana Strugala, István Molnár, Hana Šimková, Jaroslav Doležel, Ulrich Schaffrath, Matthew Barrett, Silvio Salvi, Matthew J. Moscou

**Affiliations:** 1 The Sainsbury Laboratory, University of East Anglia, Norwich Research Park, Norwich NR4 7UH, UK.; 2 NIAB, 93 Lawrence Weaver Road, Cambridge CB3 0LE, England, UK.; 3 Department of Agricultural and Food Sciences, University of Bologna, Viale G. Fanin 44, 40127 Bologna, Italy.; 4 Department of Plant Physiology, RWTH Aachen University, 52056 Aachen, Germany.; 5 Centre of the Region Haná for Biotechnological and Agricultural Research, Institute of Experimental Botany of the Czech Academy of Sciences, Šlechtitelů 31, 779 00 Olomouc, Czech Republic.; 6 Australian Tropical Herbarium, James Cook University, Smithfield 4878, Australia.

## Abstract

In the evolution of land plants, the plant immune system has experienced expansion in immune receptor and signaling pathways. Lineage-specific expansions have been observed in diverse gene families that are potentially involved in immunity but lack causal association. Here, we show that *Rps8*-mediated resistance in barley to the pathogen *Puccinia striiformis* f. sp. *tritici* (wheat stripe rust) is conferred by a genetic module: *Pur1* and *Exo70FX12*, which are together necessary and sufficient. *Pur1* encodes a leucine-rich repeat receptor kinase and is the ortholog of rice *Xa21*, and Exo70FX12 belongs to the Poales-specific Exo70FX clade. The Exo70FX clade emerged after the divergence of the Bromeliaceae and Poaceae and comprises from 2 to 75 members in sequenced grasses. These results demonstrate the requirement of a lineage-specific Exo70FX12 in Pur1-mediated immunity and suggest that the Exo70FX clade may have evolved a specialized role in receptor kinase signaling.

## Teaser

The wheat stripe rust resistance gene *Rps8* is encoded by two genes: a receptor kinase and Poales-specific Exo70.

## INTRODUCTION

Throughout evolution, plants have been exposed to diverse microbes, a subset of which are pathogenic. Recognition of pathogens by plants is mediated by two major classes of immune receptors, which are classified by the spatiotemporal properties of the interaction (*1*). The plant immune response requires pathogen detection, either at the cell boundary by membrane-localized extracellular receptors or within the cell by intracellular receptors (*1*). Pathogens facilitate infection through the synthesis and deployment of diverse effector molecules that manipulate their host to the benefit of the pathogen (*2*). This can include overcoming and suppressing immunity, directing the flow of nutrients to the pathogen, and altering the host to facilitate the pathogen’s life cycle (*3*). Effectors are translocated to the plant cell and, depending on the effector, can localize to the apoplast, the cytosol, or other subcellular compartments.

Most of the intracellular immune receptors belong to the nucleotide-binding, leucine-rich repeat (NLR) class of receptor. The intracellular defense response is typically more rapid and intense than extracellular-based responses (*4*) and is associated with lineage-specific effector recognition. Extracellular recognition occurs at the boundary to the cell, generally via membrane-bound receptor proteins (RPs) or receptor kinase (RK) proteins, which are classified based on whether the receptor has an integrated kinase domain for downstream signaling (*5*). RP and RK proteins are composed of an N-terminal exogenous binding domain and a transmembrane domain, and RK carries a C-terminal cytoplasmic kinase domain. Extracellular immune receptors recognize non-self molecular patterns associated with microbial activity via direct binding to epitopes such as the bacterial flagellin or chitin (*6*). In these examples, the recognized epitopes are conserved across a wide range of pathogens (e.g., fungal chitin), thus permitting a single receptor to provide immunity against a related group of pathogens. A subset of extracellular immune receptors recognize lineage-specific epitopes, such as Xa21 (*7*, *8*) and Stb6 (*9*), by peptides specific to *Xanthomonas* spp. and *Zymoseptoria tritici*, respectively.

Membrane-bound RPs are further categorized by the exogenous receptor domain at the N terminus of the protein (*10*). Classes of ectodomains are specialized towards particular classes of ligand such as leucine-rich repeat (LRR) domains, which primarily associate with peptide-derived ligands (*11*). LRR-RK proteins are the largest single class of RK proteins in plants (*12*). The RK/Pelle-encoding genes of *Arabidopsis thaliana* and rice have been classified into more than 60 families, of which 15 families were LRR-RK specific. The LRR-XII subfamily is markedly expanded in rice (>100 members) compared to *A. thaliana* (10 members) and contains several of the most studied plant defense LRR-RK genes, including *FLAGELLIN-SENSITIVE 2* (*FLS2*) (*13*) and *EFR* (*14*) in dicotyledonous plants and *Xa21* in the Oryzoideae (*8*). FLS2 recognizes a 22–amino acid epitope (flg22) derived from bacterial flagellin (*15*), and *EF-TU RECEPTOR* (*EFR*) recognizes an 18–amino acid peptide fragment (elf18) of the bacterial elongation factor EF-Tu, whereas Xa21 recognizes a 21–amino acid tyrosine-sulfated peptide fragment (RaxX21-sY) derived from RaxX (*7*), a type 1 secreted peptide of 61 residues that is hypothesized to have effector activity as a mimic of plant growth hormones (*16*). Extensive work in *A. thaliana* has shown that after ligand perception and binding, the protein-ligand complex is able to form a heterodimer with the LRR-II type RK BRI1-ASSOCIATED RECEPTOR KINASE (BAK1) (*17*, *18*), which interacts with both the extracellular bound ligand and the intracellular membrane-bound components of FLS2 (*19*). BAK1 is the coreceptor of EFR (*14*, *20*), and the BAK1 homolog OsSOMATIC EMBRYOGENESIS RECEPTOR-LIKE KINASE2 (OsSERK2) is required for Xa21 signaling, suggesting an ancestral requirement of the LRR-XII subfamily (*21*).

The Pucciniales are an order of obligate biotrophic fungal pathogens and causal agents of rust diseases on a wide variety of host plants (*22*). Particularly relevant to the cereal crops are *Puccinia striiformis* (stripe rust), *Puccinia triticina* (leaf rust), and *Puccinia graminis* (stem rust) (*23*). *P. striiformis* is found in all major wheat-growing areas and contributes to an average global yield loss of approximately 2% (*23*). *P. striiformis* has a complex five-stage life cycle, although only the uredinial stage directly affects cereal production (*24*). *P. striiformis* exhibits host specialization, being divided into *formae speciales* such that isolates derived from wheat (*P. striiformis* f. sp. *tritici*; *Pst*) do not routinely infect barley, whereas isolates derived from barley (*P. striiformis* f. sp. *hordei*) do not routinely infect wheat (*25*). This host specialization shows evidence of durability, as *Pst* has been endemic for over 60 years in Australia, but has not undergone a host jump to barley despite both crops being grown in the same regions (*26*). Screening of diverse barley accessions has found that *Pst* is capable of infecting and reproducing on a small subset of barley accessions: primarily landraces and wild barley, as well as the hypersusceptible accession SusPtrit (*27*). Using a diverse panel of barley accessions, we previously identified three *R* gene loci designated *Rps6*, *Rps7*, and *Rps8* as contributing to the nonadapted status of barley to *Pst* (*27*–*29*). *Rps8* was mapped to the long arm of chromosome 4H using a mapping population derived from SusPtrit × Golden Promise (SxGP) (*27*, *30*). In this work, we fine-mapped *Rps8* to a 936-kb locus on chromosome 4H, which encompasses a presence/absence variation across barley accessions. Forward genetic screens and transgenic complementation demonstrate that resistance is conferred by a genetic module including *Pur1* and *Exo70FX12*, which are each necessary and together sufficient for *Rps8*-mediated resistance.

## RESULTS

### Fine mapping of *Rps8* resolves the locus to a 936-kb region

**Fig. 1.**
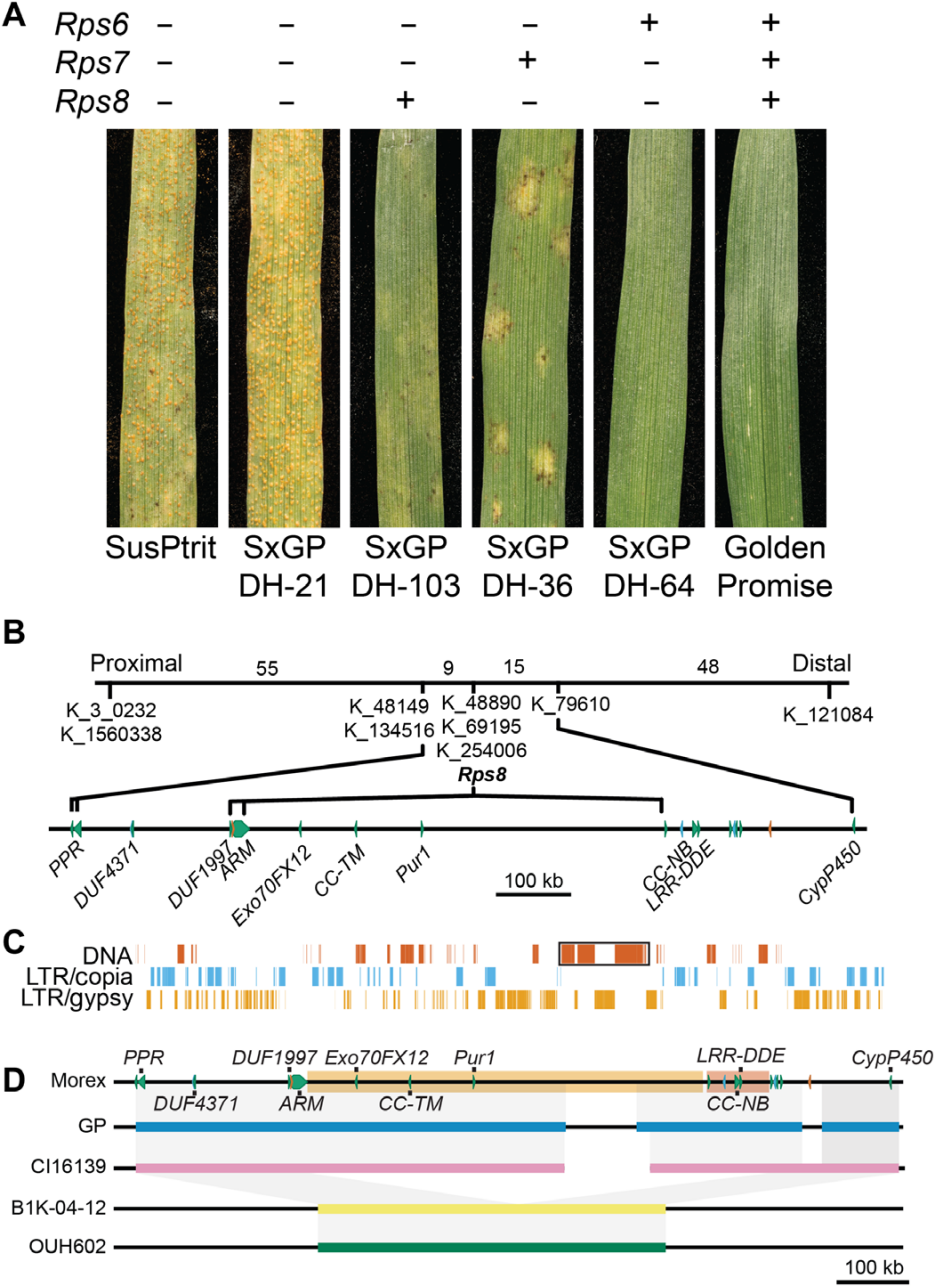
*Rps8* is associated with the presence of a 546-kb InDel. (**A**) First leaf of SxGP doubled-haploid population accessions indicating the presence (+) or absence (−) of *Rps6*, *Rps7*, and *Rps8*. (**B**) High-resolution recombination screen mapped *Rps8* to a 0.5 cM genetic interval spanning 936 kb in Morex. Genes include *DUF4371*, *DUF1997*, *ARM*, *Exo70FX12*, *LRR-RK* (*Pur1*), two *BTB/POZ*, fragmented *NLR* (*CC-NB* and *LRR-DDE*), *ZnF*, *Myb*, and several unknown and transposon-derived genes. Numbers in the genetic interval are recombinant individuals. (**C**) Repetitive content of the *Rps8* region. Tandem array of the 5′ repeat region of a CACTA DNA transposon interrupted by long-terminal repeat (LTR) retrotransposons (black box). (**D**) *Rps8* functional haplotypes are conserved, encompassing a 546-kb InDel. Genomes include Morex (*Rps8*), Golden Promise (blue; *Rps8*), CI16139 (pink; *Rps8*), and wild barley accessions B1K-04-12 (yellow) and OUH602 (green; *rps8*). A large (190 kb), repetitive region is a breakpoint in the assembly of the locus in CI16139 and Golden Promise. A 546-kb InDel (light orange) encompasses five nonrepetitive genes: *Exo70FX12*, *Pur1*, *CC-NB*, *LRR-DDE*, and *CC-TM*. A second 86-kb deletion is highlighted in a dark orange box. Colored solid lines indicate assembled contigs, whereas black lines indicate flanking or ambiguous sequence (excluding Morex).

**Fig. 2.**
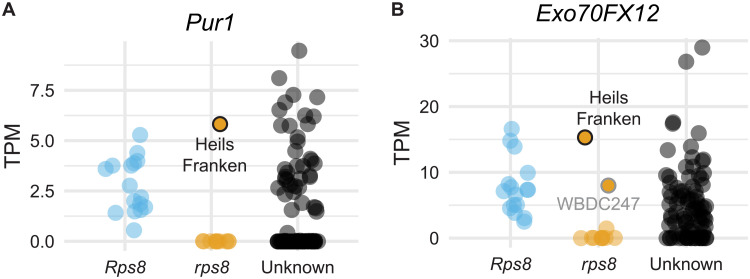
Expression level polymorphism in Pur1 and Exo70FX12 is correlated with the presence of a functional *Rps8* haplotype. (**A** and **B**) Expression level polymorphism in *Pur1* and *Exo70FX12* is correlated with the presence of a functional *Rps8* haplotype. Association of presence or absence of *Rps8* in diverse barley accessions and leaf RNAseq for *Pur1* (*HORVU.MOREX.r3.4HG0407750.1*) and *Exo70FX12* (*HORVU.MOREX.r3.4HG0407730.1*). The expression level polymorphism in *Exo70FX12* and *Pur1* is predictive for *Rps8*-mediated resistance except in the barley accessions Heils Franken and WBDC247.

Three resistance genes, *Rps6*, *Rps7*, and *Rps8*, contribute to the nonadapted status of barley to the fungal pathogen *P. striiformis* f. sp. *tritici* (*Pst*; wheat stripe rust) (Fig. 1A) (*27*). Using positional cloning, we mapped *Rps8* to a 0.5 cM genetic interval (9216 gametes) (Fig. 1B and fig. S1). This interval corresponds to 936 kb in the genome of the reference accession Morex, which carries a functional haplotype of *Rps8* (*31*) (Fig. 1, B to D). Protein-encoding genes in the *Rps8* region include *DUF4371*, *DUF1997*, Armadillo-repeat (*ARM*), Exocyst subunit *Exo70* (*Exo70FX12*), leucine-rich repeat, transmembrane, RP kinase [*Puccinia striiformis* RK 1 (*Pur1*)], two *BTB/POZ* genes, a fragmented *NLR* (*CC-NB* and *LRR-DDE*), a zinc-finger transcription factor (*ZnF*), a Myb transcription factor (*Myb*), and several unknown and transposon-derived proteins (Fig. 1B and data file S1). Individual genes in the interval were evaluated on the basis of genetic, genomic, and transcriptomic data. Twelve genes were expressed in at least one tissue type, and six genes were found to be expressed in leaves at detectable levels: *DUF1997*, *ARM*, *Exo70FX12*, *Pur1*, *ZnF*, and *Myb* transcription factors (figs. S2 and S3 and data file S1). Using association transcriptomics, we found that for most accessions, expression of *Pur1* and *Exo70FX12* was predictive of *Rps8* and *rps8* haplotypes (Fig. 2). Exceptions included the barley accessions Heils Franken and WBDC247. These results indicate that for most accessions, *Exo70FX12* and *Pur1* have an expression level polymorphism correlated with the presence of a functional *Rps8* haplotype.

### Natural variation identifies a loss-of-function mutant in *Exo70FX12*

We hypothesized that the expression polymorphism in *Exo70FX12* and *Pur1* was due to structural variation in the *Rps8* locus between resistant and susceptible haplotypes. Comparison of genomic regions encompassing *Rps8* in Morex (*Rps8*) (*31*), Golden Promise (*Rps8*) (*32*), and CI 16139 (*Rps8*) found high sequence identity (Fig. 1D). A large (190 kb), repetitive region identified in Morex was a breakpoint in the assembly of the *Rps8* locus in CI 16139 and Golden Promise (Fig. 1D). In contrast, the wild barley accessions B1K-04-12 (*33*) and OUH602 (*rps8*) (*34*) carry a 546-kb deletion that encompasses five nonrepetitive genes: *Exo70FX12*, *Pur1*, *CC-NB*, *LRR-DDE*, and *CC-TM* (Fig. 1, B and D). Reevaluation of RNA sequencing (RNA-seq) data from a panel of 109 barley accessions found that *Pur1* and *Exo70FX12* were expressed in 56 accessions and absent in 53 accessions (Fig. 2, fig. S2, and data file S2). Haplotype analysis found 15 Pur1 and 10 Exo70FX12 protein variants, which together represent 19 unique haplotypes expressing *Pur1* and *Exo70FX12*. Most accessions (*N* = 33) expressing *Pur1* and *Exo70FX12* had an identical protein sequence with the Morex haplotype. All other haplotypes were identified in wild barley except for the landrace Heils Franken, which carries a single nonsynonymous polymorphism in *Exo70FX12* (CDS G813A, protein E271K). While the barley accession Heils Franken is resistant to *Pst*, the underlying genetic architecture for this resistance is unknown. Genetic mapping in F_2_ and BC_1_ populations found no linkage to *Rps8*, indicating that Heils Franken lacks a functional haplotype of *Rps8* (figs. S4 to S6). Whole-genome sequencing, alignment, and variant calling of barley accession Heils Franken found that the only variant in annotated protein-encoding genes in the *Rps8* region was the previously identified nonsynonymous polymorphism in *Exo70FX12* creating an E271K modification. This suggests that the *Exo70FX12* is likely required for *Rps8*-mediated resistance.

### A forward genetic screen identifies loss-of-function mutations in *Pur1* and *Exo70FX12*

**Fig. 3.**
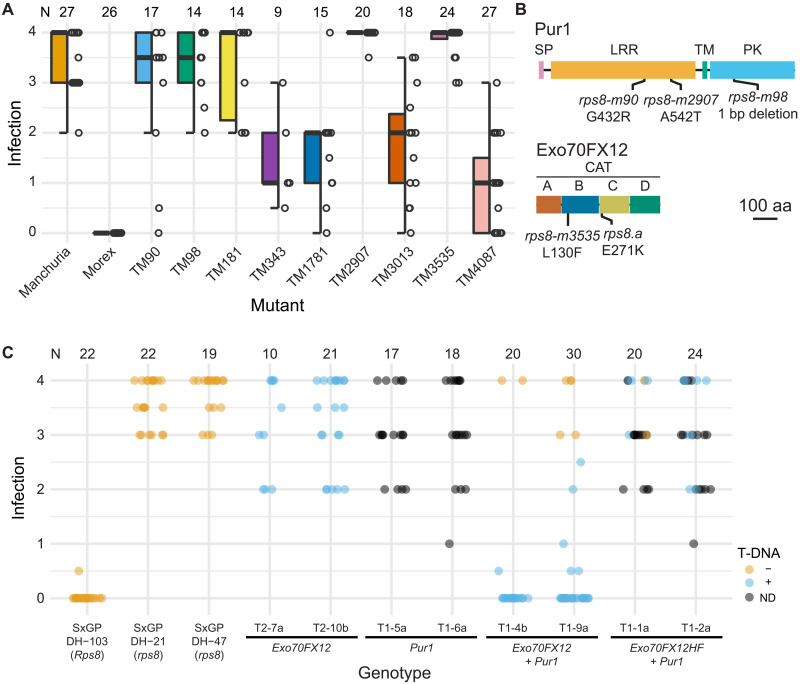
*Rps8*-mediated resistance requires both *Pur1* and *Exo70FX12*. (**A**) Infection phenotypes of *Rps8*-mediated resistance mutants using *Pst* isolate 16/035. Panel shows boxplots, individual data points, and total number of evaluated plants (*N*) over three biological replicates. (**B**) Positions of sodium azide–induced mutations and natural variation in barley cv. Heils Franken (*rps8.a*) within Exo70FX12 and Pur1 primary structure. Subdomain structure of Exo70FX is based on conservation with yeast Exo70. The domain structure of Pur1 is based on InterProScan. (**C**) Infection phenotypes for independent single-copy transformants of barley cv. SxGP DH-47 expressing *Exo70FX12*, *Pur1*, *Exo70FX12* and *Pur1*, and *Exo70FX12HF* (Heils Franken allele) and *Pur1*, under their native promoters and terminators. The presence (blue) or absence (orange) of the T-DNA was determined using a quantitative polymerase chain reaction (qPCR)–based assay. When not determined (ND), data points are in black. Inoculations were performed using *Pst* isolate 16/035 and scored at 14 days after inoculation, *N* shows the number of evaluated seedlings.

We initiated a forward genetic screen to identify genes underpinning *Rps8*-mediated resistance using an advanced mutant population in the reference accession Morex (TM population) (*35*), which harbors *Rps8* in isolation from other loci providing *Pst* resistance (*27*). Evaluation of 1526 M_6_ families with *Pst* isolate 16/035 identified 37 putative mutants (table S1). Single seed descent and subsequent reevaluation of putative mutants with *Pst* isolate 16/035 confirmed that nine mutants were susceptible to *Pst* (Fig. 3A). Leaf RNA-seq of the mutants found three independent mutations in the *Pur1* CDS: *rps8-TM90* (CDS G1409A, protein G432R) and *rps8-TM2907* (CDS G1624A, protein A542T) harboring missense mutations in the LRR encoding region and *rps8-TM98* carrying a deletion in the protein kinase encoding region that generates a truncated kinase domain (CDS 2504) (Fig. 3B). In addition, the mutant line *rps8-TM3535* carries a missense mutation in the *Exo70FX12* CDS (CDS C388T, protein L130F) (Fig. 3B). Analysis of RNA-seq from five additional susceptible mutant lines did not identify any variation in genes at the *Rps8* locus, indicating that these mutations likely occur at other loci. To assess the specificity of susceptibility in these mutants, we challenged them with the nonadapted pathogen *P. triticina* (wheat leaf rust). Barley accession Morex and all tested mutants were resistant to *P. triticina* (fig. S7). To confirm the genetic relationship of *rps8* mutants, pairwise crosses were performed between a subset of mutants at the *Rps8* locus and F_1_ progeny inoculated with *Pst* isolate 16/035. The mutants TM90 and TM98 were found to belong to the same complementation group, and TM3535 was independent (fig. S8). These results confirm that both *Pur1* and *Exo70FX12* are necessary for *Rps8*-mediated resistance.

### *Pur1* and *Exo70FX12* are necessary and sufficient to confer *Rps8*-mediated resistance

We hypothesized that transgenic complementation of *Pur1* and *Exo70FX12* in isolation would be insufficient to confer *Rps8*-mediated resistance and that this would require the presence of both genes. We performed *Agrobacterium*-based transformation using the *Pst*-susceptible accession SxGP DH-47 and a T-DNA construct encoding the native genomic region encompassing *Exo70FX12* (fig. S9). We recovered eight T_1_ transgenic families and found no association with the presence/absence of the T-DNA and segregating phenotypes when inoculated with *Pst* isolate 16/035 (fig. S10A). This suggests that *Exo70FX12* is insufficient to confer *Rps8*-mediated resistance. To confirm that the *Exo70FX12* transgene was functional, we crossed homozygous T_2_ from T_1_-10b, T_1_-7b, and T_1_-14a lineages with the susceptible mutant TM3535 that carries a single nonsynonymous mutation in *Exo70FX12*. While homozygous advanced T_2_ and T_3_ lineages were susceptible, all F_1_ progeny derived from crosses with TM3535 were resistant to *Pst* isolate 16/035 (fig. S10B). Therefore, *Exo70FX12* is sufficient to complement the mutant TM3535 but insufficient to confer *Rps8*-mediated resistance when transformed in SxGP DH-47.

To test whether expression of both *Pur1* and *Exo70FX12* is required to confer *Rps8*-mediated resistance, we transformed the *Pst*-susceptible accession SxGP DH-47 with T-DNA constructs that natively express *Pur1* and *Exo70FX12* individually, *Pur1* and *Exo70FX12* together, and the *Pur1* with the *Exo70FX12* allele from Heils Franken (fig. S9). T_1_ families containing single T-DNA inserts, T_2_ families homozygous for natively expressed *Exo70FX12*, and controls (Manchuria, Morex, SxGP DH-47) were inoculated with *Pst* isolate 16/035 (Fig. 3C). T_1_ families carrying the *Pur1* expressed under its native promoter and T_2_ families homozygous for the *Exo70FX12* expressed under its native promoter were susceptible to *Pst* isolate 16/035, as were T_1_ families carrying *Pur1* and *Exo70FX12* allele from Heils Franken (Fig. 3C). In contrast, T_1_ families carrying a T-DNA containing both *Pur1* and *Exo70FX12* under their native promoters conferred resistance that cosegregated with the presence of the T-DNA (Fig. 3C). An extended set of transgenic T_1_ families supported these observations (fig. S11). Collectively, these results demonstrate that *Pur1* and *Exo70FX12* together are necessary and sufficient to confer *Rps8*-mediated resistance.

### *Pur1* belongs to the LRR-XII subfamily of RK and is the ortholog of *Xa21*

**Fig. 4.**
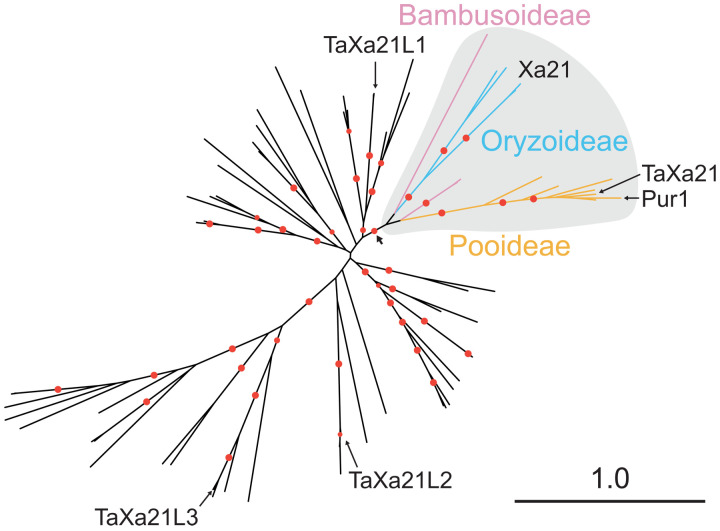
*Pur1* is the ortholog of *Xa21*. Maximum likelihood unrooted phylogenetic tree based on 69 full-length RK proteins from the Xa21/Pur1 clade and putative Xa21 homologs from 24 Poaceae species (data file S5). Color coding on the subtree indicates the tribe origin of branches [Bambusoideae (pink), Oryzoideae (blue), and Pooideae (orange)]. Red dots indicate bootstrap support greater than 80%. The basal branch on the Xa21 subtree indicated by an arrow is supported at 95%. Scale indicates 1.0 substitution per site.

LRR-RK proteins are the predominant class of extracellular recognition receptors (*6*). *Pur1* encodes a 1080–amino acid protein with a domain structure of signal peptide (1 to 16 amino acids), 24 imperfect 24–amino acid LRRs (74 to 652 amino acids), transmembrane (67 to 695 amino acids), juxtamembrane (702 to 726 amino acids), and protein kinase (727 to 1038 amino acids) (fig. S12). Sequence analysis found that Pur1 belongs to the LRR-XII subfamily (*36*) that includes the immune receptors Xa21 (*8*), FLS2 (*13*), and EFR (*14*). To ascertain the relationship of Pur1 relative to other LRR-XII members, we constructed a phylogenetic tree based on full-length LRR-XII subfamily protein sequences from eight grass species (fig. S13 and data file S3). Pur1 was found in the Xa21 subclade (data S4). Subclade analysis using putative homologs in 24 additional grass species found that Pur1 and Xa21 are orthologs (Fig. 4 and data file S5). In agreement with previous phylogenetic analysis, three wheat Xa21-like RKs, TaXa21L1, TaXa21L2, and TaXa21L3, are not in the Xa21 subclade (*37*). In contrast, the RK-encoding gene *TaXa21* is within the Xa21 subclade and is the wheat ortholog of Pur1 (*38*). *TaXa21* was identified through induced gene expression after high temperature and was associated with a quantitative reduction in *Pst* uredinia production in a temperature-dependent manner based on virus-based silencing (*38*). While no further genetic or functional characterization was performed, these results indicate that orthologs of *Xa21* in barley and wheat both contribute to immunity to *Pst*. Pur1 shares 55% (590 of 1080) amino acid identity to Xa21 and is largely conserved in domain structure except for an additional LRR domain between the seventh and eighth LRR domains of Xa21 and the presence of a 19–amino acid insertion in the kinase domain of Pur1 (fig. S12). Loss-of-function mutation *rps8-TM90* (G432R) is in a conserved residue (G414), whereas *rps8-TM2907* (A542T) is variable between Pur1 (A542) and Xa21 (V542). To ascertain the position of these mutations relative to the predicted protein structure, we generated structural predictions of the LRR and kinase domains using AlphaFold (*39*) (movie S1). We found that Pur1 G432 is located on the LRR backbone, whereas A542 is on the inner concave surface of the LRR ectodomain. This suggests that the A542T mutation likely affects the structure of Pur1, whereas G432R may affect the interaction of Pur1 with its ligand or coreceptor.

### *Exo70FX12* belongs to the Poales-specific *Exo70FX* subfamily

**Fig. 5.**
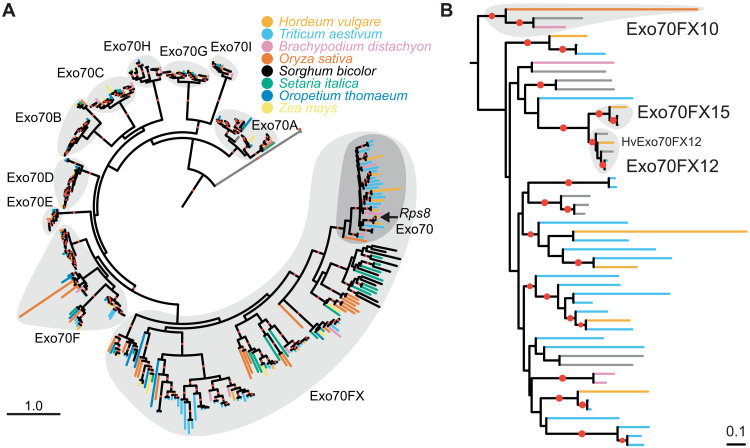
*Rps8* Exo70 belongs to the Poales-specific Exo70FX subfamily. (**A**) Rooted maximum likelihood (ML) phylogenetic tree based on 365 full-length Exo70 proteins from barley (*H. vulgare*), wheat (*Triticum aestivum*), purple false brome (*Brachypodium distachyon*), rice (*O. sativa*), *O. thomaeum*, maize (*Z. mays*), foxtail millet (*S. italica*), and sorghum (*S. bicolor*) (data file S7). Structure-guided multiple sequence alignment was performed using MAFFT DASH with yeast, human, mouse, and *A. thaliana* Exo70A1 proteins. Rooted using yeast, human, and mouse Exo70 as outgroups. The 10 present Exo70 subfamilies (A, B, C, D, E, F, G, H, I, and FX) are distinguished with distinct light gray regions, and the Exo70FX10/11/12/15 clades are further highlighted in dark gray. *Rps8 Exo70* encodes HvExo70FX12. Red dots indicate bootstrap support greater than 80%. Scale indicates 1.0 substitution per site. (**B**) Rooted ML phylogenetic tree using 51 full-length Exo70 proteins from the Exo70FX10/11/12/15 clades and putative Exo70FX homologs from 13 Pooideae species (data file S4). Rooted using the Exo70FX10 clade as an outgroup. Red dots indicate bootstrap support greater than 80%. Scale indicates 0.1 substitutions per site.

Exo70, along with Sec3, Sec5, Sec6, Sec8, Sec10, Sec15, and Exo84, comprise the octameric Exocyst complex (*40*, *41*), which is involved in tethering secretory vesicles to the plasma membrane in concert with soluble *N*-ethylmaleimide–sensitive factor attachment protein receptor (SNARE) proteins (*42*). In vascular plants, Exo70s are highly expanded, spanning 11 clades (A, B, C, D, E, F, G, H, I, J, and FX) (*43*–*45*). In angiosperms, the majority of clades are conserved between monocot and dicot plants. The Exo70FX clade is unique to monocots and members exhibit substantial sequence divergence. To determine the phylogenetic relationship of *Exo70FX12*, we generated a structure-guided phylogenetic tree for Exo70 proteins from eight Poaceae species (Fig. 5A and data file S6). Exo70FX12 is a member of the Exo70FX clade. Exo70FX12 is embedded within Exo70FX11 subclade (Fig. 5B and data file S7). *Exo70FX11* members belong to a single multigene locus on chromosome 2H, whereas *Exo70FX12* is a single copy gene on chromosome 4H.

Previous work has shown that the Exo70FX clade is present in all evaluated Poaceae (grasses) species (*44*). Our initial analysis encompassed genomes from eight Poaceae species. To ascertain the origin of the Exo70FX clade, we identified Exo70 clade members in the recently sequenced genomes of *Streptochaeta angustifolia* (*46*) and *Pharus latifolius* (*47*), which represent critical species at the boundary of the Poaceae family. In addition, we included *Ananas comosus* (pineapple; Poales, Bromeliaceae) (*48*) and *Musa acuminata* (banana; Zingiberales, Musaceae) (*49*). Phylogenetic analysis of Exo70 from these species with barley, rice, foxtail millet, and maize Exo70 found conservation in all non-Exo70FX clades (table S2). In contrast, while the Exo70FX clade was present in *P. latifolius* and *S. angustifolia*, it was absent in pineapple and banana. Therefore, the Exo70FX clade emerged after the divergence of the most recent ancestor of the Bromeliaceae-Poaceae.

### *Exo70FX12* emerged in the Triticeae and exists as a *trans*-species presence/absence variation

To delineate the evolutionary origin of *Exo70FX12*, we searched sequenced genomes and de novo assembled transcriptomes from species in the Bambusoideae, Oryzoideae, and Pooideae. Phylogenetic analysis with putative nonredundant *Exo70FX12* homologs within the genera *Aegilops*, *Triticum*, *Agropyron*, and *Hordeum* found that translocation of this gene from the *Exo70FX11* locus occurred in a common ancestor of the Triticeae (Fig. 5B). The *Exo70FX11* clade includes the only characterized Exo70FX member, *HvExo70FX11b*, that when transiently silenced was found to contribute to penetration resistance against powdery mildew (*50*). Our analysis uncovered a second previously unidentified Exo70FX in barley that is absent in the reference genome but present in the barley accession Barke and located at a distinct locus on chromosome 2H. As this gene appears to be a unique transposition of the gene family, we designated it *Exo70FX15*. The *Exo70FX15* clade is sister to the *Exo70FX12* clade and was found in the genera *Aegilops*, *Triticum*, and *Hordeum*. *Exo70FX12* was found in all sequenced *Triticum* B subgenomes (*N* = 19), *Aegilops speltoides* (two accessions; *N* = 3), *Aegilops sharonensis* (two accessions; *N* = 16), *Aegilops tauschii* (four accessions; *N* = 16), and *Agropyron cristatum* (one accession; *N* = 2); *Pur1* and *Exo70FX12* co-occurred in most evaluated accessions (data S4). We conclude that *Pur1* is ancient, with an origin before diversification of the BOP clade, whereas *Exo70FX12* and *Exo70FX15* are recent innovations within the Triticeae, both derived from the *Exo70FX11* locus.

### The Exo70FX family is experiencing substantial expansion, diversification, and N-terminal truncation

The substantial diversification on Exo70FX prompted an examination of the conservation of subdomains within this family. In yeast, cryo–electron microscopy (cryo-EM) of the Exocyst complex has found that Exo70 is composed of five subdomains: CorEx, CAT-A, CAT-B, CAT-C, and CAT-D (*51*). Exo70 CorEx interacts with Exo84 CorEx to create the first-level heterodimer, which then forms a four-helix bundle with the CorEx subdomains of Sec10 and Sec15 (*51*). Exo70 CAT-C and CAT-D interface with Sec5 CAT-B and CAT-C. Plasma membrane interaction via phospholipid binding is mediated by CAT-D (*52*). To assess variation in Exo70 subdomain composition, we assessed amino acid coverage of Exo70 clades within the multiple sequence alignment relative to *Saccharomyces cerevisiae* Exo70. We found several clade-specific presence/absence of CorEx and CAT-A subdomains (fig. S14). Exo70 clades Exo70A, Exo70B, Exo70C, Exo70F, Exo70G, and Exo70I show retention of all five subdomains, whereas Exo70D, Exo70E, and Exo70H have reduced N-terminal coverage, suggesting loss of the CorEx subdomain. The Exo70FX clade has a unique coverage pattern, indicating that most members have lost CorEx and CAT-A subdomains. This observation was supported by a reduction in protein length in most members in the Exo70FX clade (fig. S15). Subdomain analysis of Exo70FX12 found that it lacked the canonical CorEx subdomain and has a truncated CAT-A subdomain. Structural prediction of Exo70FX12 using AlphaFold predicts an extended rod composed of alpha helices (movie S2). CAT-B, CAT-C, and CAT-D subdomains are complete, whereas the CorEx subdomain is absent and the CAT-A subdomain is substantially truncated. Exo70FX12 mutation L130 is buried in the CAT-B subdomain, whereas E271 is solvent-exposed in the CAT-C subdomain. In summary, these results show that the Exo70FX clade is experiencing substantial species-specific expansion, high sequence divergence as compared to non-Exo70FX clades, and loss of the CorEx and CAT-A subdomain.

## DISCUSSION

We previously identified three *R* gene loci designated *Rps6*, *Rps7*, and *Rps8*, which provide resistance to the nonadapted pathogen *Pst* across a variety of cultivated barley accessions (*27*–*29*). Fine mapping of *Rps8* to a 936-kb locus on chromosome 4H identified a presence/absence variation across diverse barley accessions. Using a forward genetic screen, natural variation, and transgenic complementation, we found that *Rps8*-mediated resistance is conferred by a genetic module: *Pur1* and *Exo70FX12*, which are together necessary and required for *Rps8*-mediated resistance. *Pur1* belongs to the RK/Pelle LRR-XII subfamily, and phylogenetic analysis of grasses found that it is the barley ortholog of rice *Xa21*. *Exo70FX12* belongs to the Exo70FX clade that emerged after the divergence of the most recent ancestor of the Bromeliaceae-Poaceae. *Exo70FX12* is a single-copy Triticeae-specific gene that originated via transposition of a single member of the multicopy *Exo70FX11* locus. The Exo70FX clade exhibits several features that contrast it with other clades such as loss of N-terminal CorEx and CAT-A subdomains, substantial sequence divergence among family members, and intraspecific presence/absence variation.

In green plants, transmembrane receptors and plant-specific kinases have experienced a massive expansion (*53*). Among these receptors, LRR-RK proteins are highly expanded and are the predominant class of extracellular recognition receptors (*12*, *54*). LRR-RK proteins are involved in regulating growth, abiotic stress responses, and extracellular recognition of non-self molecules (*12*). The LRR-RK gene family experiences species-specific and subclade-specific selective pressure, varying between purifying and expansion/diversifying, in a manner consistent with subclade-specific roles in growth and stress responses, as well as immunity (*54*). The LRR-XII subfamily is associated with defense, and several well-characterized LRR-RK genes were involved in immunity including FLS2, EFR, and Xa21. Phylogenetic analysis of the RK Pelle LRR-XII subfamily found that *Pur1* and *Xa21* are orthologs, sharing a common ancestor before the radiation of the Bambusoideae, Oryzoideae, and Pooideae [43 million years (Ma) ago, CI: 27 to 58 Ma ago (*55*)]. Expression of the wheat ortholog, *TaXa21*, was found to be induced after heat treatment, and transient virus-mediated silencing conferred a quantitative increase in *Pst* uredinia production in a temperature-dependent manner (*38*). Therefore, the recognition of *Pst* is likely an ancestral state before the radiation of the Triticeae and implicates the orthogroup in resistance to adapted and nonadapted pathogens of wheat and barley, respectively. Among the RK LRR-XII subfamily in plants, *Pi65* and *Pur1* are functionally validated family members that confer resistance to a fungal pathogen (*56*). FLS2 and EFR recognize well-conserved epitopes found in many bacteria, flagellin, and EF-Tu, respectively, whereas Xa21 recognizes a tyrosine-sulfated peptide derived from RaxX in *Xanthomonas oryzae* (*7*). While the ligand recognized by Pur1 is unknown, this work supports the previously proposed hypothesis that the large expansion of LRR-XII genes in monocots is a shift toward recognizing ligands that are specific to a particular pathogen, in contrast with the characterized LRR-XII subfamily members of dicots (*12*).

The octomeric Exocyst complex is found in fungi, metazoa, and plants with a primary role in localization and tethering of secretory vesicles to the cell membrane (*41*, *52*). Genetic, functional, and structural analysis in yeast established that the Exocyst complex is composed of eight proteins: Sec3, Sec5, Sec6, Sec8, Sec10, Sec15, Exo70, and Exo84 (*40*, *41*). Within the complex, Exo70 interacts with Exo84, Sec10, and Sec15 via an N-terminal CorEx domain and with Sec5 via C-terminal CAT-C and CAT-D domains (*51*). Exo70 is an important hub for the Exocyst complex, interacting with the plasma membrane component (phosphatidylinositol 4,5-bisphosphate), SNARE proteins, RHO guanosine triphosphatase (GTPase) proteins, Rab GTPase proteins, and Arp2/3 via its C-terminal domains (*52*). Fusion with the membrane and secretion itself involve a variety of additional proteins, primarily components of the SNARE complex (*52*). While animal and fungal genomes encode a single copy of the Exo70 gene, plants have evolved numerous copies that can be further divided into 11 clades: Exo70A, Exo70B, Exo70C, Exo70D, Exo70E, Exo70F, Exo70G, Exo70H, Exo70I, Exo70J, and Exo70FX (*43*–*45*). These clades are nonredundant and exhibit clear evidence of subfunctionalization: They are expressed in different tissue types, localize to different membrane domains, and carry different cargoes (*57*, *58*). While clades Exo70A, Exo70B, Exo70C, Exo70D, Exo70E, and Exo70G are well conserved, the remaining clades exhibit interspecific variation. Exo70I is required for the establishment of mycorrhizal symbioses and is not present in species that do not form these associations (*59*). Exo70J is unique to legume species (*43*). Exo70H, which is involved in trichome development and defense, is expanded in dicots (six to eight copies) relative to monocots (one to three copies) (*44*, *60*, *61*). Conversely, Exo70F is expanded in monocots, while Exo70FX is unique to monocots (*44*).

Using recently released genomes within the Poales, phylogenetic analysis of the Exo70FX clade found that the clade emerged after the divergence of the most recent common ancestor of Bromeliaceae-Poaceae. *Exo70FX12* is a member of the Exo70FX clade. To date, only one member of the Exo70FX clade has been characterized, *Exo70FX11b*, which was found to contribute toward penetration resistance against powdery mildew when transiently silenced (*50*). In barley, the *Exo70FX11* locus is located on chromosome 2H and contains six family members that show evidence of subfunctionalization based on tissue-specific expression. *Exo70FX12* is derived from the *Exo70FX11* locus, which translocated to chromosome 4H before the radiation of the Triticeae as orthologs were found in *A. cristatum*, *Ae. tauschii*, *Ae. speltoides*, and *Triticum aestivum* B genome. Several lines of evidence indicate that the Exo70FX clade is experiencing a different evolutionary trajectory as compared to other Exo70 families. First, the Exo70FX clade experiences substantial sequence divergence, observed by long branch lengths on the phylogenetic tree, and extensive interspecific copy number variation compared to other clades. Second, of the 10 Exo70 clades in grasses, only Exo70D and Exo70FX experience variable loss of the N-terminal region. For Exo70D, this is restricted to the CorEx domain, whereas Exo70FX proteins routinely lack CorEx and/or CAT-A domains, which normally interact with Exo84 to form an antiparallel zipper that facilitates integration into the octomeric Exocyst complex (*51*). Third, we identified intraspecific variation for novel members of the Exo70FX clade, including *Exo70FX12* and *Exo70FX15*. This contrasts with other clades where interspecific variation is low, and no evidence exists for intraspecific presence/absence variation. Our current analysis has been restricted to the Pooideae, although we hypothesize that gene family expansion mediated by translocation may be common within other grass lineages for the Exo70FX clade. Fourth, Exo70F and Exo70FX are the only two clades identified as integrated domains in NLRs (*62*), suggesting a role in immunity or as the target of pathogen effectors. To the latter hypothesis, the rice blast effector AVR-Pii directly interacts with OsExo70F2 and OsExo70F3, and the interaction with OsExo70F3 is required for resistance mediated by the rice CC-NB-LRR pair Pii-1 and Pii-2 (*63*). Last, *Exo70FX12* is required for *Rps8*-mediated resistance in conjunction with *Pur1*, suggesting that *Exo70FX12* has evolved a specialized role in the plant immune response. The Exo70FX11 family members are insufficient to complement this function, despite several members being expressed in leaf alongside *Exo70FX12*. Collectively, this suggests that the Exo70FX clade is experiencing intense selective pressure that may have its origins in adaptive evolution.

Using a forward genetic screen on a barley accession carrying *Rps8* in isolation, we identified three *pur1* mutants, one *exo70FX12* mutant, and five additional loss-of-function mutants. Sequence analysis of these five loss-of-function mutants found no mutations in *Pur1* and *Exo70FX12*, indicating that these lines carry mutations in loci *Required for* Rps8*-mediated resistance* (*Rsr*). Further work will be required to identify these *Rsr* genes. Extensive forward and reverse genetic screens on *FLS2*, *EFR*, and *Xa21* have identified genes regulating their maturation, translocation, signaling, and degradation (*64*). These screens have uncovered proteins involved at different stages in the secretory pathway including the endoplasmic reticulum, the Golgi apparatus, and the trans-Golgi network (*65*–*68*). The final stage of protein transport to the plasma membrane involves the tethering of vesicles to the membrane mediated by the Exocyst complex, followed by the fusion of the secretory vesicle catalyzed by the SNARE complex (*52*). The role of the Exocyst complex in membrane receptor translocation was uncovered through reverse genetics in *A. thaliana* (*69*, *70*). Initially, mutants in *AtEXO70B2* were found to have greater susceptibility to a range of pathogens (*69*) and later found to be compromised in early responses to the elicitors flg22, elf18, chitin, and Pep1 (*70*). *A. thaliana* EXO70B1 is also required for flg22-mediated early immune responses, with other elicitors not tested (*70*). Further work found that AtEXO70B1 and AtEXO70B2 are essential for proper FLS2 homeostasis and trafficking to the membrane (*71*). EXO70B1 and EXO70B2 were found to directly interact with FLS2 and hetero-oligomerize (*71*). This role in membrane receptor signaling is likely conserved in monocots, as mutants in *OsExo70B1* have increased susceptibility to rice blast (*Magnaporthe oryzae*), and OsExo70B1 interacts directly with the chitin receptor CHITIN ELICITOR RECEPTOR KINASE 1 (CERK1) (*72*). Analogous to FLS2, Pur1 requires an Exo70 for proper functioning. The specialized role of Exo70FX12 in barley contrasts with the conserved role of Exo70B family members for maintaining diverse RK abundance at the plasma membrane (*71*, *72*). As the Exo70B clade is conserved in all angiosperms (*44*) and expressed in all cell layers of barley leaves, this indicates that Exo70FX12’s role in Pur1 signaling is distinct from the activities of Exo70B.

In plants, immune receptors such as RKs and NLRs experience expansion and diversification, likely due to the selective pressure exerted by plant pathogens. For the Exo70 gene family, tissue-specific expression facilitating regulation of secretion likely underscored the early expansion of the gene family in plants (*57*, *58*). Compared to other Exo70 clades, the rapid expansion and diversity of the Exo70FX clade may indicate that further subfunctionalization and/or neofunctionalization has occurred. Given that only two *Exo70FX* members have been characterized (*50*), and both are involved in defense, it seems likely that this expansion is connected to an overall role in immunity. While the molecular function of the Exo70FX12 is unclear, its requirement in LRR-RK signaling implicates several possible models. In the first model, Exo70FX12 localizes Pur1 to an appropriate domain of the plasma membrane with the involvement of other members of the Exocyst complex. Exo70FX12 would retain its capacity to interact with the plasma membrane and Exocyst complex members. For the second model, Exo70FX12 localizes Pur1 to the plasma membrane in an unconventional (Exocyst-independent) manner. Our third model is that Exo70FX12 is not involved with localization of Pur1 and is involved in signal transduction, perhaps as a scaffold to facilitate the interaction with other proteins. Different Exo70 clades are known to participate in diverse biological processes such as autophagy (*60*, *73*) and mycorrhization (*59*), which may or may not depend on their ancestral role as an Exocyst complex member. This work has established a genetic function for Exo70FX12 in RK-mediated immunity and suggests that the Exo70FX clade may have evolved a specialized role in plant immunity.

Genetic modules are rare in plant immunity and hitherto involve NLR intracellular immune receptors. Examples include the NLR Prf that monitors the conformational status of Pto, a protein kinase (*74*, *75*) and the paired NLRs RPP2A/RPP2B (*76*). The unique configuration of Pur1/Exo70FX12 reflects the potential role of the Exo70FX clade in RK signaling. Whether *Exo70FX12* functions in *Rps8*-mediated resistance within the context of exocytosis, either in an Exocyst-dependent or -independent manner, or is instead involved in signal transduction, is unknown. Future work on elucidating the molecular mechanism of Exo70FX12 will illuminate the significance of the Exo70FX clades’ emergence and expansion in the Poaceae and further our understanding of RK-mediated immunity in grasses.

## MATERIALS AND METHODS

### Plant and fungal materials

Barley accessions were obtained from diverse sources (data file S2). SxGP DH-21, SxGP DH-47, and SxGP DH-103 are individuals from the SxGP doubled-haploid population, which was provided by R. Niks (Wageningen University, Netherlands) (*30*). All plants were propagated through single seed descent before experimentation. *Pst* isolates 08/21 and 16/035 and *P. triticina* isolate 20/018 were collected in 2008, 2016, and 2020, respectively, in the United Kingdom and maintained at the National Institute of Agricultural Botany (NIAB). *Pst* and *P. triticina* isolates were increased on susceptible wheat cultivars, collected, and stored at 6°C.

### Pathogen assays

*Puccinia striiformis* f. sp. *tritici* (*Pst*) inoculations were carried out by sowing seeds in groups of eight seeds per family, with four families spaced equidistantly around the rim of each 1-liter pot of John Innes peat-based compost. Plants were grown at 18°C in the day and at 11°C at night using a 16-hour light/8-hour dark cycle in a controlled environment chamber at NIAB, with lighting provided by metal halide bulbs (Philips MASTER HPI-T Plus 400W/645 E40). Barley seedlings were inoculated at 12 days after sowing, where first leaves were fully expanded and the second leaf was just beginning to emerge. Urediniospores of *Pst* were suspended in talcum powder, at a 1:16 ratio of urediniospores to talcum powder based on weight. Compressed air was used to inoculate seedlings on a spinning platform. After inoculation, seedlings were placed in a sealed bag and stored at 8°C for 48 hours (incubation period) to increase humidity for successful germination of urediniospores. Subsequently, plants were returned to the growth chamber for the optimal development of *Pst* and phenotyped at 14 days after inoculation. Plants were scored using a nine-point scale from 0 to 4, with increments of 0.5, for chlorosis (discoloration) and infection (pustule formation) (*77*). The scale indicates the percentage of leaf area affected by the corresponding phenotype where a score of 0 indicates asymptomatic leaves, i.e., no chlorosis, browning, or pustules, and a score of 4 indicates leaves showing the respective phenotype over 100% of the surface area. *P. triticina* inoculations were carried out in a similar manner to *Pst* with modifications including a 72-hour incubation period, postincubation period growing conditions of 22°C (day) and 12°C (night), and phenotypic assessment at 12 days after inoculation.

### Microscopic phenotyping

Fixation, staining, and quantification of *Pst* in barley leaves were carried out according to Dawson *et al.* (*77*). Briefly, barley leaves were collected at 14 days after inoculation, placed in 1.0 M KOH with surfactant (Silwet L-77), and incubated at 37°C overnight (12 to 16 hours). Leaves were washed three times in 50 mM tris at pH 7.5. Leaf tissue was incubated in 1.0 ml of wheat germ agglutinin–fluorescein isothiocyanate (WGA-FITC) solution [WGA-FITC (20 μg/ml) in 50 mM tris at pH 7.5] overnight, mounted, and observed under blue-light excitation on a fluorescence microscope with a green fluorescent protein filter. pCOL estimates the percent of leaf colonized, and pPUST estimates the percent of leaf harboring pustules. Phenotyping was performed by evaluating the leaf surface in equally sized, adjacent portions. Within each field of view (FOV), the colonization of *Pst* was estimated to be less than 15%, between 15 and 50%, or greater than 50% of the FOV area and given scores of 0, 0.5, or 1, respectively. The final pCOL score was determined by averaging these scores based on the total number of FOVs evaluated and ranged from 0 to 100%. pPUST was evaluated in a similar manner but for the clustering pattern of *Pst* pustules. A 5× objective with an FOV of 2.72 mm × 2.04 mm was used.

### Genetic analysis

DNA was extracted from leaf tissue using a 96-well plate-based cetyltrimethyl ammonium bromide (CTAB)-based method (*27*). Genotyping was performed using KASP assays at the John Innes Centre Genotyping Facility (Norwich, UK). Genetic maps for the Manchuria × Heils Franken F_2_ and BC_1_ populations were developed using markers spanning the consensus genetic map of barley approximately every 10 cM (data file S8). Genetic distances were computed using MapManager QTX (v20) using the Kosambi function. Two-point linkage tests (plotRF; R v4.1.1 and R/qtl v1.4.8-1) were used to evaluate genetic map integrity. Composite interval mapping was performed using QTLcartographer v1.17j using five background markers, a step size of 1 cM, and forward-background selection of background markers. Significance thresholds at α at 0.05 were determined using 1000 permutations with resampling of background markers.

The *Rps8* recombination screen was performed using 4608 SxGP DH-21 × SxGP DH-103 F_2_ using the markers K1_1398 and K1_1470 identifying 127 recombinants. Marker saturation in the *Rps8* region was performed using the genomes of Barke, Bowman, Morex, and Haruna Nijo and RNAseq data from SusPtrit and Golden Promise. The *Rps8* region was delimited to the flanking markers K_4819 and K_079610_445 and located in the barley genome using NCBI BLAST^+^ v2.2.31. Informative markers were applied to all recombinants derived from the recombination screens. A minimum of 16 individuals from F_2:3_ families were independently assessed using *Pst* isolate 16/035.

### Long-range assembly of CI 16139 chromosome 4H

Chromosome flow sorting of CI 16139 chromosome 4H was performed using the methods described by Doležel *et al.* (*78*), and chromosomal high molecular weight (HMW) DNA was prepared as described in Thind *et al.* (*79*) (fig. S16). Chicago Dovetail sequencing of the chromosome was performed by Dovetail Genomics (Santa Cruz, CA, USA), with initial assembly in Meraculous (v2.0.3) and final scaffolding in HiRise. Briefly, Chicago libraries were generated by in vitro chromatin reconstitution using 250 ng of HMW DNA. After fixation with formaldehyde, chromatin was digested with Mbo I, biotinylated nucleotides were added to 5′ overhangs, and proximity ligation was performed. After reversal of cross-linking, the DNA was treated to remove biotin and sheared to approximately 400 bp. A sequencing library was generated using NEBNext Ultra enzymes (New England Biolabs) and Illumina-compatible adapters and sequenced using Illumina HighSeq X to produce 229 million paired-end reads, which gave 95× coverage for chromosome 4H. An initial assembly using Meraculous had length 521.7 Mb on 57,043 scaffolds. The HiRise assembly had 527.17 Mb on 1702 scaffolds.

### Genomic analyses

A GATK variant calling pipeline was used to identify variants in the barley accession Heils Franken relative to the reference genome (accession Morex). Briefly, raw reads were trimmed with Trimmomatic (v0.39) using parameters ILLUMINACLIP:TruSeq3-PE.fa:2:30:10, LEADING:5, TRAILING:5, SLIDINGWINDOW:4:15, and MINLEN:36. Alignment of reads onto the barley genome was performed with bwa mem (0.7.12-r1039) using parameters “-T 0 -M -R.” GATK (v4.2.0.0) was used to sort (default parameters), mark duplicates (default parameters), perform variant calling using HaplotypeCaller with parameters “--dont-use-soft-clipped-bases --standard-min-confidence-threshold-for-calling 20,” and perform variant filtering using VariantFiltration with parameters QUAL > 40, DP > 9, and QD > 20.0, which were based on assessment of the dataset distributions. The impact of variants on protein encoding genes was assessed with snpEff (v5.0d) with default parameters. Comparison of genomic regions encompassing the *Rps8* locus was performed using dotplots (dottup EMBOSS suite) in Geneious Prime (v2021.2.2) (www.geneious.com). Repetitive DNA was annotated using RepeatMasker (v4.1.2-p1) (www.repeatmasker.org) using Dfam with RepBase for RepeatMasker (RBRM) (v3.2), Triticeae Repeat Sequenc (TREP) (v19), and rmblastn (v2.11.0+) databases.

### Transcriptomic analysis

RNA was extracted from the first and second leaves of 10-day-old plants. Tissue was harvested, frozen in liquid nitrogen, and ground to a fine powder using a mortar and pestle with grinding sand at −80°C. Ground tissue was suspended in TRI RNA isolation reagent and allowed to incubate for 5 min at room temperature before centrifugation at 12,000*g* to pellet the lysate. The supernatant was recovered and mixed with chloroform. This mixture was incubated at room temperature for 15 min before the phases were separated by centrifugation, and the lighter phase was recovered. Nucleic acids were precipitated from the lighter phase with isopropanol, pelleted via centrifugation, then washed with 75% ethanol, and resuspended in water. After extraction, RNA was purified and assessed for quality (*28*). RNA libraries were constructed using Illumina TruSeq RNA library preparation (Illumina; RS-122-2001) and sequenced using 100- or 150-bp paired-end reads (Novogene). Read trimming was performed with Trimmomatic (v0.39) with the following parameters: ILLUMINACLIP:2:30:10 using TruSeq3 paired-end adapters, LEADING:5, TRAILING:5, SLIDINGWINDOW:4:15, and MINLEN:36. Spliced alignment of RNA-seq reads to a DNA template was performed using hisat2 v2.2.1 using default parameters. Alignment of RNA-seq reads to a complementary DNA template was performed using bwa mem (0.7.12-r1039) using default parameters. The QKgenome pipeline was used to identify sequence variation in *Pur1* and *Exo70FX12*. Briefly, reads were aligned to the 936-kb *Rps8* region of the sequenced Morex genome using hisat2 (v2.2.1) with the default parameters. Samtools v1.11 was used to compress and sort reads. The number of reads mapping to each nucleotide position within the locus was calculated using bedtools genomecov. QKgenome_conversion.py integrates variant calling and coverage to identify variants in annotated genes with coverage greater than or equal to 10 reads. De novo transcriptomes were assembled using Trinity (v2.4.0) using default parameters. For tissue-specific expression, RNA-seq data were processed using Trimmomatic, as described above, and kallisto (0.46.0) using the barley-predicted transcriptome (version 3) to determine expression level (transcripts per million) using 100 bootstraps.

### Construct development and plant transformation

*Exo70FX12* coding sequence and its native promoter and terminator were amplified from barley Golden Promise genomic DNA (gDNA) using Phusion High-Fidelity DNA Polymerase (NEB). Polymerase chain reaction (PCR) primers were designed 2 kb upstream of the predicted start of transcription and 1.5 kb downstream of the predicted stop codon. PCR was performed by assembling a reaction mix containing 2.5 μl of Phusion Master Mix (NEB), 2.5 μl of deoxynucleotide triphosphates (dNTPs) (200 μM), 1 μl of forward and reverse primer (SH_12_p1f and SH_12_p1r; 400 nM each), 1 μl of DNA (100 ng of gDNA or 10 ng of plasmid DNA), and 0.2 μl of Phusion Taq polymerase (NEB) per reaction vessel using a Bio-Rad G-Storm GS4 thermocycler, set to cycle through: 1.5 min at 94°C, 35 repeats of 30 s at 94°C, 30 s at 50°C, 30 s per kilobase at 72°C, 5 min at 72°C, and a cooling stage of 10°C. PCR product was gel-excised and extracted before cloning into pCR-XL-2-TOPO vector (Invitrogen). Plasmids were extracted from *E. coli*–transformed cells and sequenced using Sanger sequencing (Genewiz, Oxford, UK). The complete *Exo70FX12* transcriptional unit was then assembled into pBract202 binary vector (BRACT) using the Gibson method. Briefly, fragments harboring 20-bp overlapping overhangs were PCR-amplified from the *Exo70FX12* transcriptional unit and the acceptor pBract202 vector, then purified, and mixed in an equimolar ratio (200 ng of total DNA) with 15 μl of home-made 1.33× one-step isothermal DNA assembly master mixture prepared as described in Gibson *et al.* (*80*). Reaction was incubated at 50°C for 1 hour followed by transformation into *E. coli* heat-shock competent cells. Plasmids were extracted from *E. coli*–transformed cells.

Constructs with *Pur1* and *Exo70FX12* + *Pur1* constructs were assembled using the Golden Gate cloning method. Domesticated (removal of internal Bsa I/Bbs I sites without changes in encoded amino acids) coding sequence, native promoter, and native terminator DNA parts were synthesized (Twist Bioscience) and separately cloned into Golden Gate compatible level 0 vectors. Restriction-ligation reactions (15 μl) were carried out with 1× T4 DNA ligase buffer (NEB), 1.5 μg of bovine serum albumin, 5 U of Bbs I (Thermo Fisher Scientific), 1000 U of T4 DNA ligase (NEB), 100 ng of acceptor vector, and twice the molar amount of the DNA part. Reactions were performed for 26 cycles of alternating incubations at 37°C for 3 min and 16°C for 4 min, followed by 5 min at 50°C and 5 min at 80°C to inactivate the enzymatic components. After *E. coli* transformation, corresponding transcriptional units were assembled into Golden Gate level 1 vectors by a similar restriction-ligation procedure but using Bsa I instead of Bbs I. The *Exo70FX12HF* + *Pur1* construct was generated from the *Exo70FX12* + *Pur1* construct by site-directed mutagenesis. Final constructs were subsequently assembled into pICSL4723 Golden Gate compatible binary vector (AddGene #48015) by Bbs I-T4 DNA ligase restriction-ligation reactions. All primers used for molecular cloning are described in table S3.

Assembled constructs were then introduced into *Agrobacterium tumefaciens* strain AGL1 by electroporation. Barley plant transformation was performed using the *Agrobacterium*-mediated transformation method described by Hensel and Kumlehn (*81*). T-DNA insert copy number testing was performed by iDna Genetics (Norwich, UK) by quantitative real-time PCR using the selectable marker gene *hyg*.

### Domain, subdomain, and phylogenetic analysis of the Exo70 gene family

To identify proteins containing Exo70 domains, InterProScan v5.36-75.0 using default parameters was used on predicted transcripts and annotated CDS. Proteins annotated with the Exo70 Pfam family (PF03081) were extracted. Structure-guided multiple sequence alignment of Exo70 proteins was performed with MAFFT software (v7.481) using DASH (Database of Dligned Structural Homologs) with default parameters. Exo70 structures included in the alignment were derived from *A. thaliana* Exo70A1 (PDB 4RL5), *S. cerevisiae* (yeast) Exo70 (PDB 2B1E and 5YFP), and *Mus musculus* (mouse) (PDB 2PFT). The phylogenetic tree was constructed using RAxML (v8.2.12) with the JTT amino acid substitution model, gamma model of rate heterogeneity, and 1000 bootstraps. A convergence test performed using RAxML autoMRE found convergence after 200 bootstraps. iTOL (Interactive Tree of Life) was used for phylogenetic tree visualization, and *S. cerevisiae* and *M. musculus* Exo70 were used as outgroups. For the subdomain analysis of Exo70, a domain was considered present if at least 30 total residues were present over the alignment region corresponding to that domain in *S. cerevisiae* Exo70. Protein modeling was performed with Phyre2 using normal modeling mode.

### Phylogenetic analysis of RK LRR-XII subfamily

To identify RK LRR-XII subfamily in diverse grass species, we used a hidden Markov model (HMM)–based approach developed by Lehti-Shiu and Shiu (*36*). Briefly, all kinase domain–containing proteins were identified using 38 kinase Pfam identifiers (table S4). Grass species genomes were accessed from diverse sources (data file S4). De novo assembled transcriptomes were generated for species with available leaf RNA-seq datasets (data file S2). Putative kinases were classified with HMMs based on individual kinase families using hmmalign (HMMER 3.1b2). Multiple sequence alignment was performed using kalign with default parameters. The QKphylogeny_alignment_analysis.py script was used to filter the alignment for variable sites represented in at least 20% of proteins and sequences spanning at least 40% of the alignment length (https://github.com/matthewmoscou/QKphylogeny). Maximum likelihood phylogenetic tree construction was performed using RAxML using the JTT amino acid substitution model, the gamma model of rate heterogeneity, and 1000 bootstraps. Visualization of the phylogenetic tree was performed with iTOL.

### Protein structure prediction

AlphaFold (v2.1.0) implemented as a Google Colab notebook using default parameters was used to predict protein structure. Protein sequences used include the ectodomain of Pur1 (amino acids 1 to 688) and full-length Exo70FX12. Visualization of predicted protein structure was performed using PyMOL (v2.5.2).

## Notes

View/request a protocol for this paper from *Bio-protocol*.
