# Supplementary material for: A lineage-specific Exo70 is required for receptor kinase–mediated immunity in barley
Source: Sci Adv. 2022 Jul 6;8(27):eabn7258. doi: 10.1126/sciadv.abn7258 (PMC9258809; doi:10.1126/sciadv.abn7258)
Supplement: Supplementary file 1 — Figs. S1 to S16 Tables S1 to S4 [file sciadv.abn7258_sm.pdf]

Science Advances
AAAS

Supplementary Materials for

A lineage-specific Exo70 is required for receptor kinase–mediated immunity in barley

Samuel Holden *et al.*

Corresponding author: Matthew J. Moscou, matthew.moscou@tsl.ac.uk

Sci. Adv. **8**, eabn7258 (2022)
DOI: 10.1126/sciadv.abn7258

The PDF file includes:

Figs. S1 to S16
Tables S1 to S4
Legends for movies S1 and S2
Legends for data S1 to S8

Other Supplementary Material for this manuscript includes the following:

Movies S1 and S2
Data S1 to S8

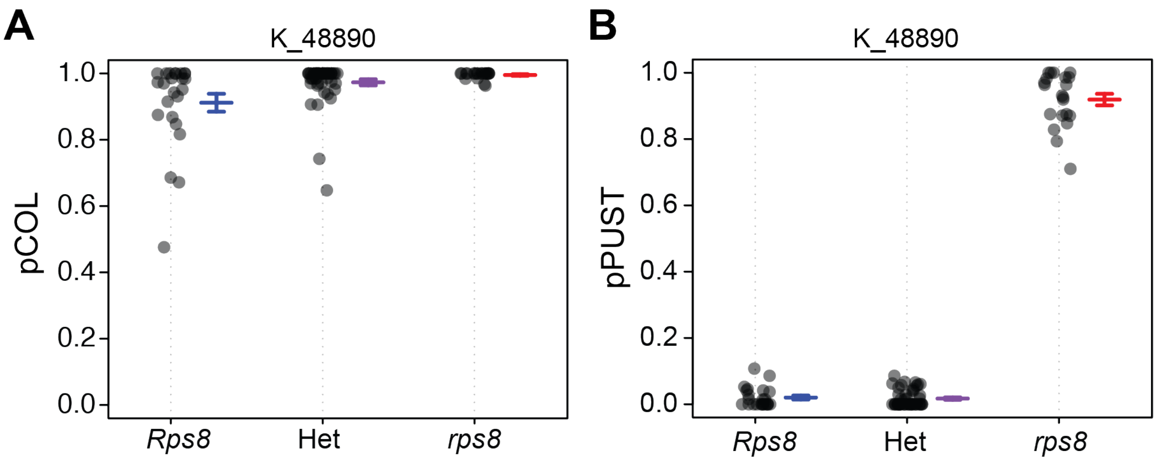

Fig. S1. *Rps8* segregates as a dominant gene. (A) Colonization (pCOL) and **(B)** pustule formation (pPUST) were assessed in a SxGP DH-21 (*rps6 rps7 rps8*) x SxGP DH-103 (*rps6 rps7 Rps8*) F_2 population inoculated with *Pst* isolate 16/035 (N=94). *Rps8* co-segregates with the KASP marker K_48890.

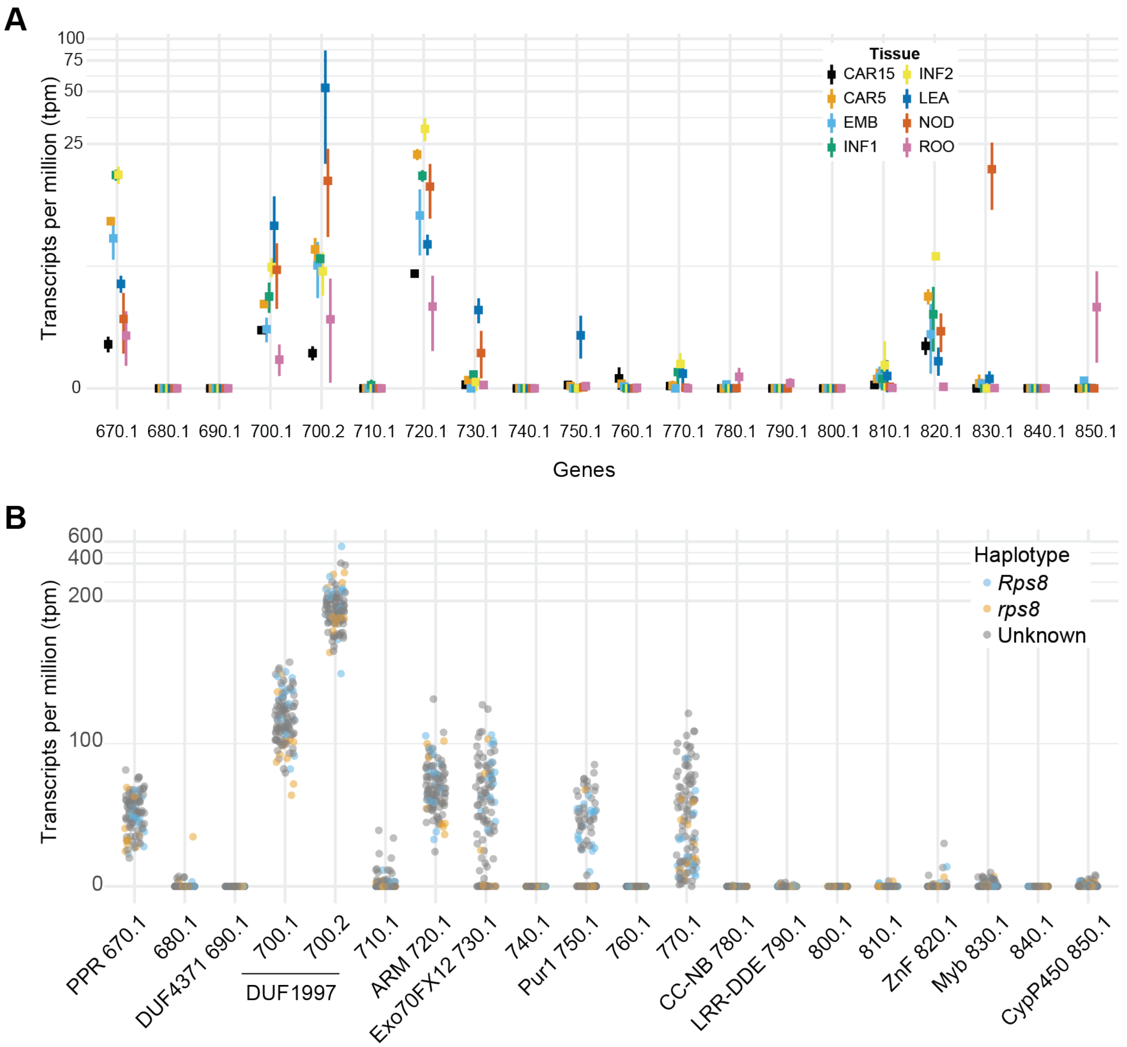

Fig. S2. Expression analysis of genes in the *Rps8* locus in eight diverse tissues. (A) RNAseq data was obtained from EMBL/ENA accession ERP001600 (*80*), trimmed, and used to estimate expression level (transcripts per million; y-axis; pseudo-log scale) based on the barley predicted transcriptome (*12*). Identifiers (x-axis) correspond to the suffix of genes within the *Rps8* locus based on the longer identifier HORVU.MOREX.r3.4H0407xxx. Mean and standard deviation are shown as square and whiskers. Color coding and order shows the tissue including early developing grain (15 days post anthesis (CAR15) and 5 (CAR5)), germinating grain (4 day) embryos (EMB), early developing inflorescences (5 (INF1) & 15 mm (INF2)), shoots from seedlings (LEA; 10 cm stage), developing tiller internodes (NOD) (six-leaf stage), and roots from seedlings (ROO; 10 cm stage). **(B)** Leaf expression level of genes in 109 barley accessions in the *Rps8* region. The y-axis shows expression level based on transcripts per million (TPM).

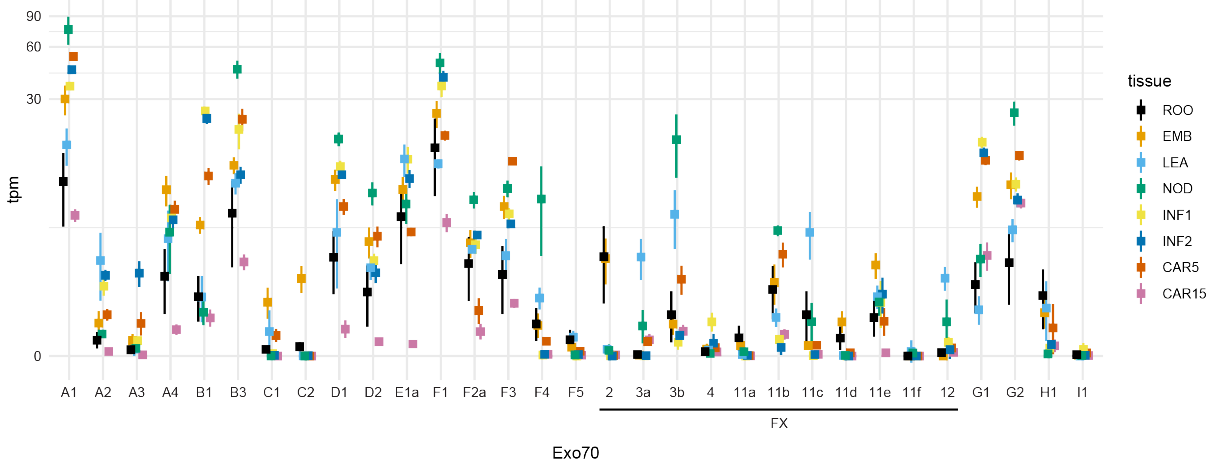

Fig. S3. Expression analysis of barley Exo70 genes in eight diverse tissues. RNAseq data was obtained from EMBL/ENA accession ERP001600 (*80*), trimmed, and used to estimate expression level (transcripts per million (tpm); y-axis; pseudo-log scale) based on the barley predicted transcriptome (*12*). Identifiers (x-axis) correspond to the suffix of Exo70 encoding genes. Mean and standard deviation are shown as a square and whiskers. Color coding and order shows the tissue including early developing grain (15 days post anthesis (CAR15) and 5 (CAR5)), germinating grain (4 day) embryos (EMB), early developing inflorescences (5 (INF1) & 15 mm (INF2)), shoots from seedlings (LEA; 10 cm stage), developing tiller internodes (NOD) (six-leaf stage), and roots from seedlings (ROO; 10 cm stage).

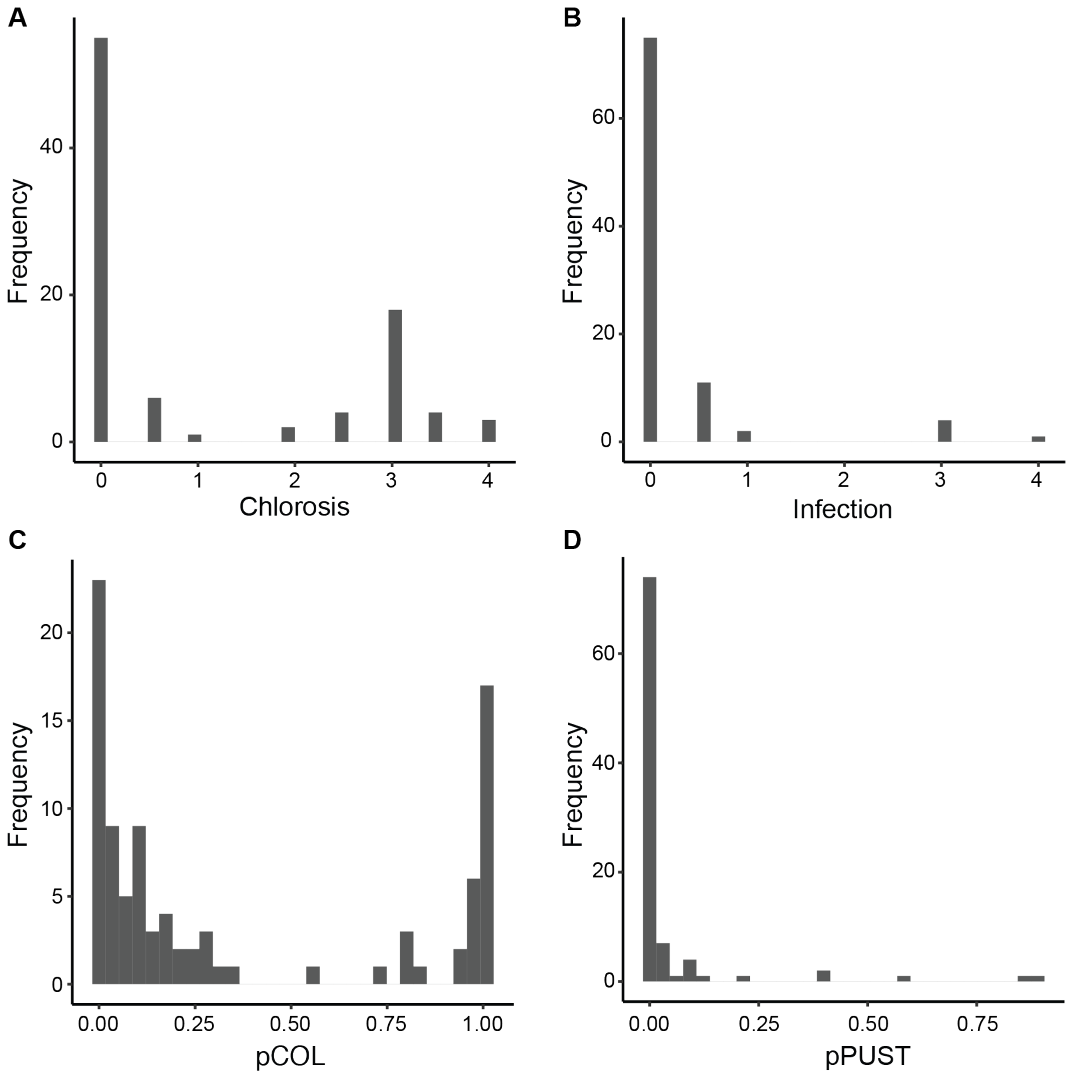

Fig. S4. Phenotypic distributions of disease traits in the Manchuria x Heils Franken F_2 population inoculated with *Pst* isolate 08/21. Histograms showing the phenotypic distributions for macroscopic **(A)** chlorosis and **(B)** infection and microscopic colonization (pCOL) **(C)** and pustule formation (pPUST) **(D)**. The population is composed of 94 F_2 individuals.

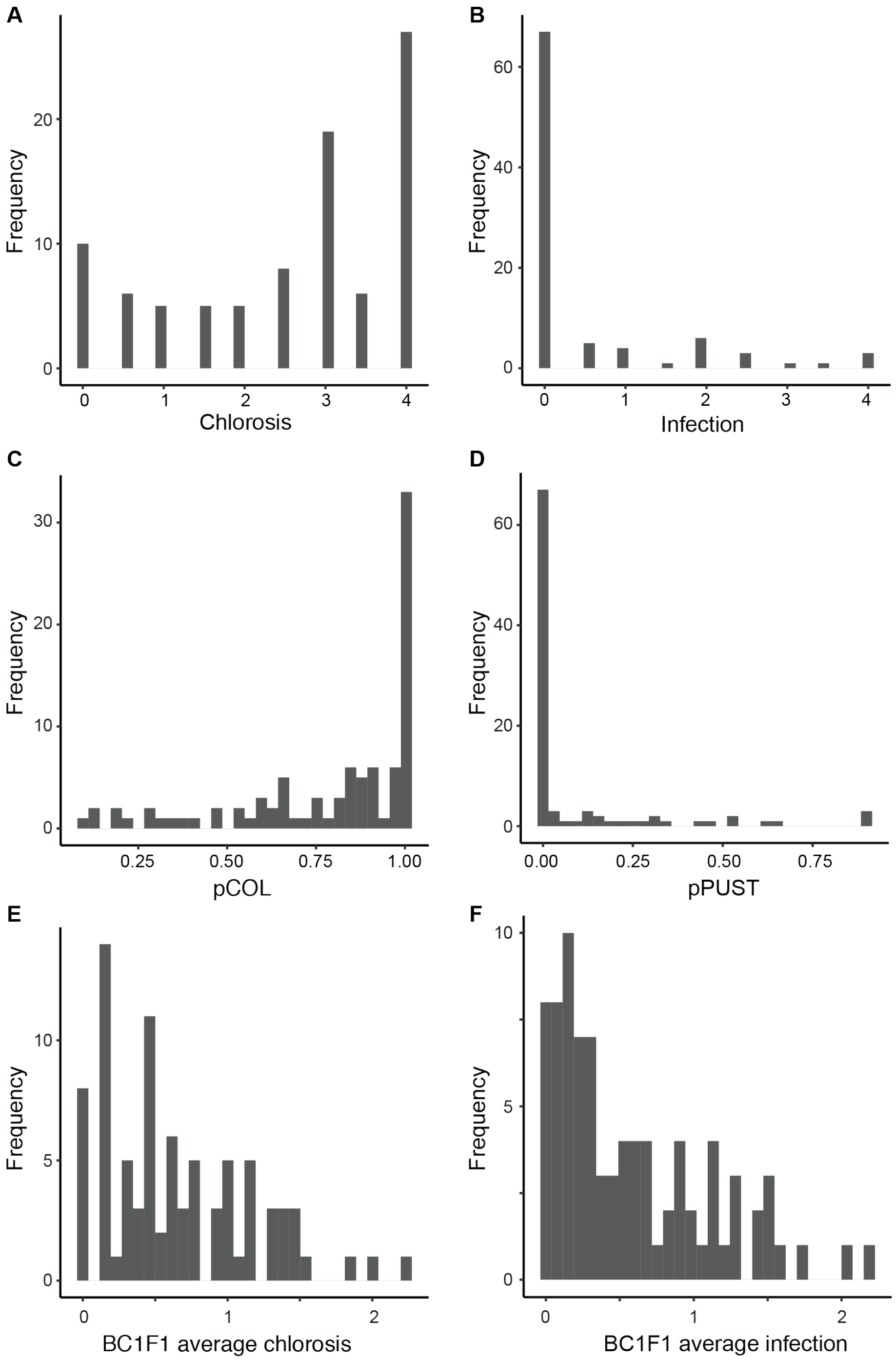

Fig. S5. Phenotypic distributions of resistance traits in the Manchuria x Heils Franken BC_1 population and BC_1F_1 progeny inoculated with *Pst* isolate 08/21. Histograms showing the phenotypic distributions for macroscopic **(A)** chlorosis and **(B)** infection and microscopic colonization (pCOL) **(C)** and pustule formation (pPUST) **(D)** of the Manchuria x Heils Franken BC_1 population (N=94), and average chlorosis **(E)** and infection **(F)** phenotypes for the Manchuria x Heils Franken BC_1F_1 population (N=85; 8 seedlings per family).

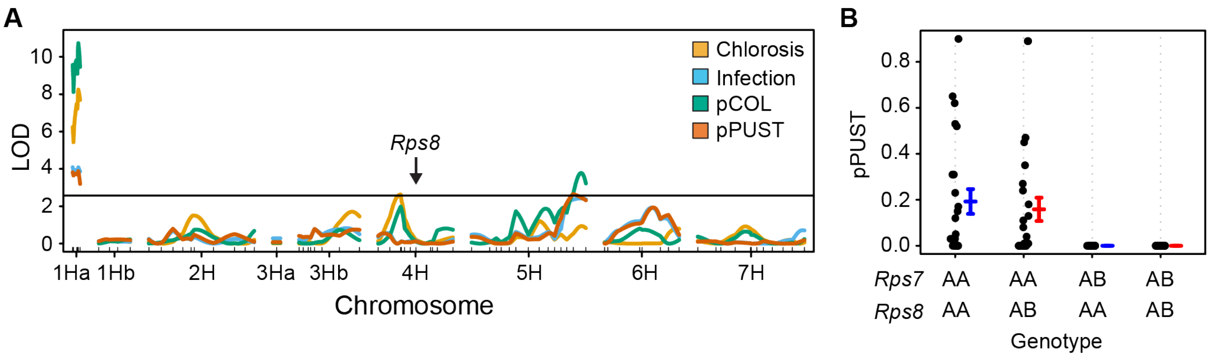

Fig. S6. Barley accession Heils Franken does not carry a functional haplotype of *Rps8*. (A) Quantitative trait locus analysis of *Pst* isolate 16/035 resistance in the Manchuria x Heils Franken BC_1 population. QTLs conferring resistance were identified on 1H, 4H, and 5H, but none overlapped with the *Rps8* locus. **(B)** Phenotype x genotype plot of the *Rps7* and *Rps8* loci based on the Manchuria x Heils Franken BC_1 population from **(A)**. Strong linkage is observed for *Rps7*, but not *Rps8* for pPUST (pustule formation).

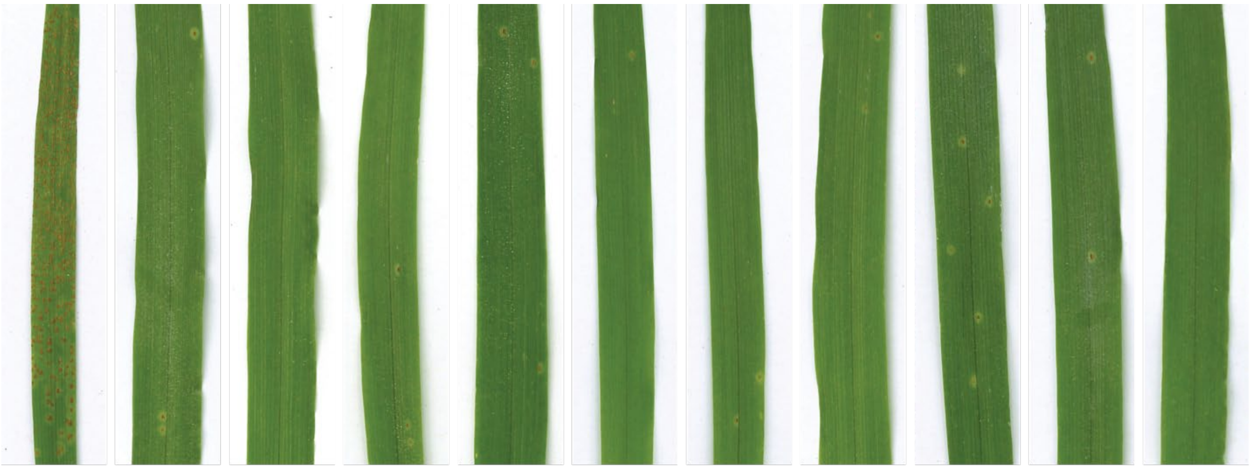

Fig. S7. *Rps8*-mediated resistance mutants maintain resistance to the non-adapted pathogen *Puccinia triticinia* (wheat leaf rust) isolate 20/018. Photographs show representative leaves. The experiment was performed with two biological replicates that included eight individual leaves per experiment. Controls include the wheat accession Fielder and barley accession Morex (*Rps8*).

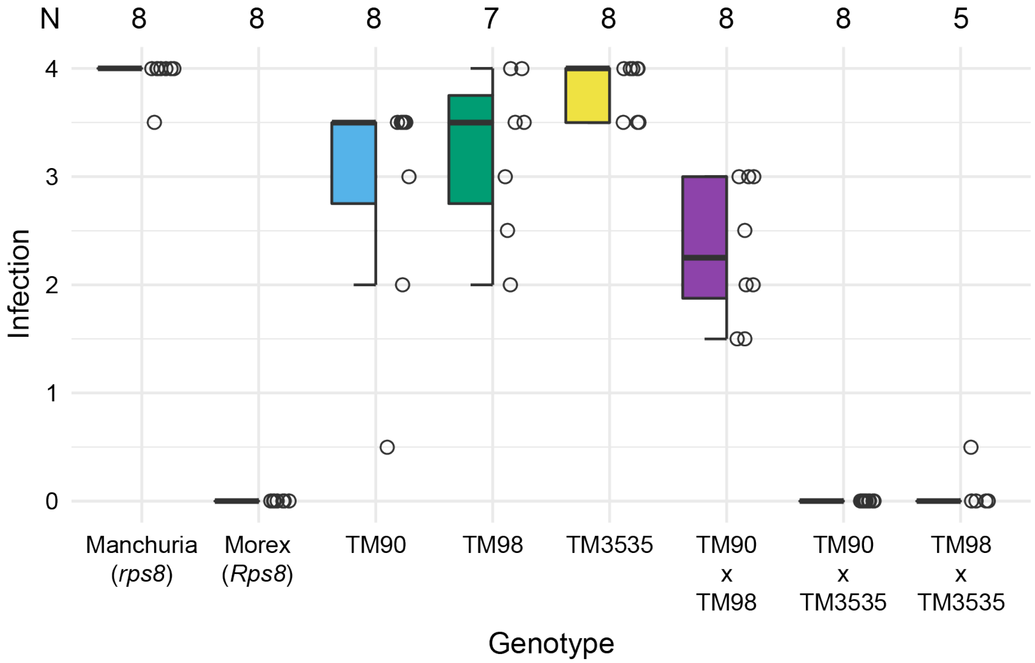

Fig. S8. Complementation tests for *rps8* mutants TM90, TM98, and TM3535. Infection phenotypes (y-axis) of confirmed mutants and pairwise F_1 progeny inoculated with *Pst* isolate 16/035. For each genotype (x-axis), boxplot and individual data points are shown from a single experiment that included F_1 individuals. Total number of evaluated individuals (N) is shown. Data for controls Manchuria (*rps8*), Morex (*Rps8*), TM90, TM98, and TM3535 is a subset of data shown in Figure 3d.

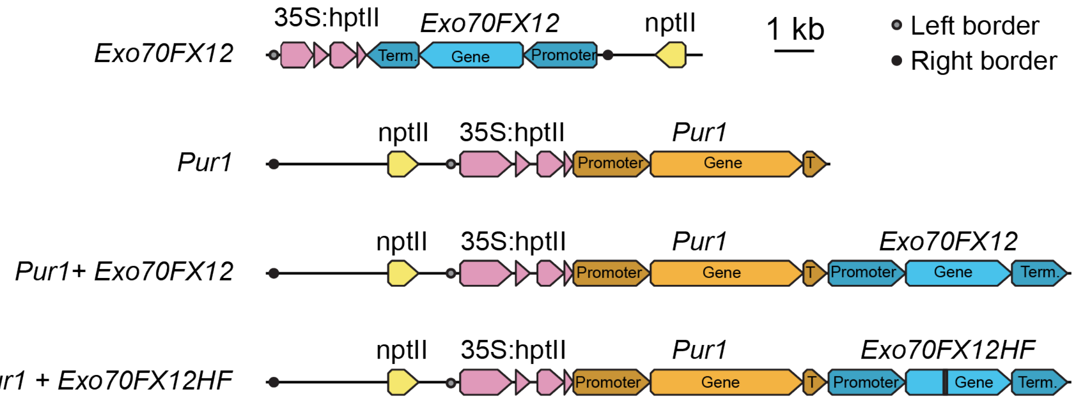

Fig. S9. Transformation constructs for candidate genes at the *Rps8* locus. The *Exo70FX12* construct was generated using a PCR fragment encompassing the native genomic context of *Exo70FX12* with approximately 2 kb promoter and 1.5 kb terminator. The constructs *Pur1* and *Pur1* + *Exo70FX12* were generated by synthesizing domesticated fragments of native genomic context with approximately 2 kb promoter and 0.6 kb (*Pur1*)/1.5 kb (*Exo70FX12*) terminator and assembled using Golden Gate cloning. *Pur1* + *Exo70FX12HF* was generated using site-directed mutagenesis using the *Pur1* + *Exo70FX12* construct as template, recreating the Heils Franken allele for *Exo70FX12*. Promoter, gene, and terminator (Term. or T) regions are indicated. All constructs used nptII as a bacterial selectable marker (shown in yellow) and hptII driven by the 35S Cauliflower Mosaic Virus (35S) promoter for plant selection during transformation (shown in pink). Left and right T-DNA borders are shown with filled grey and black circles, respectively.

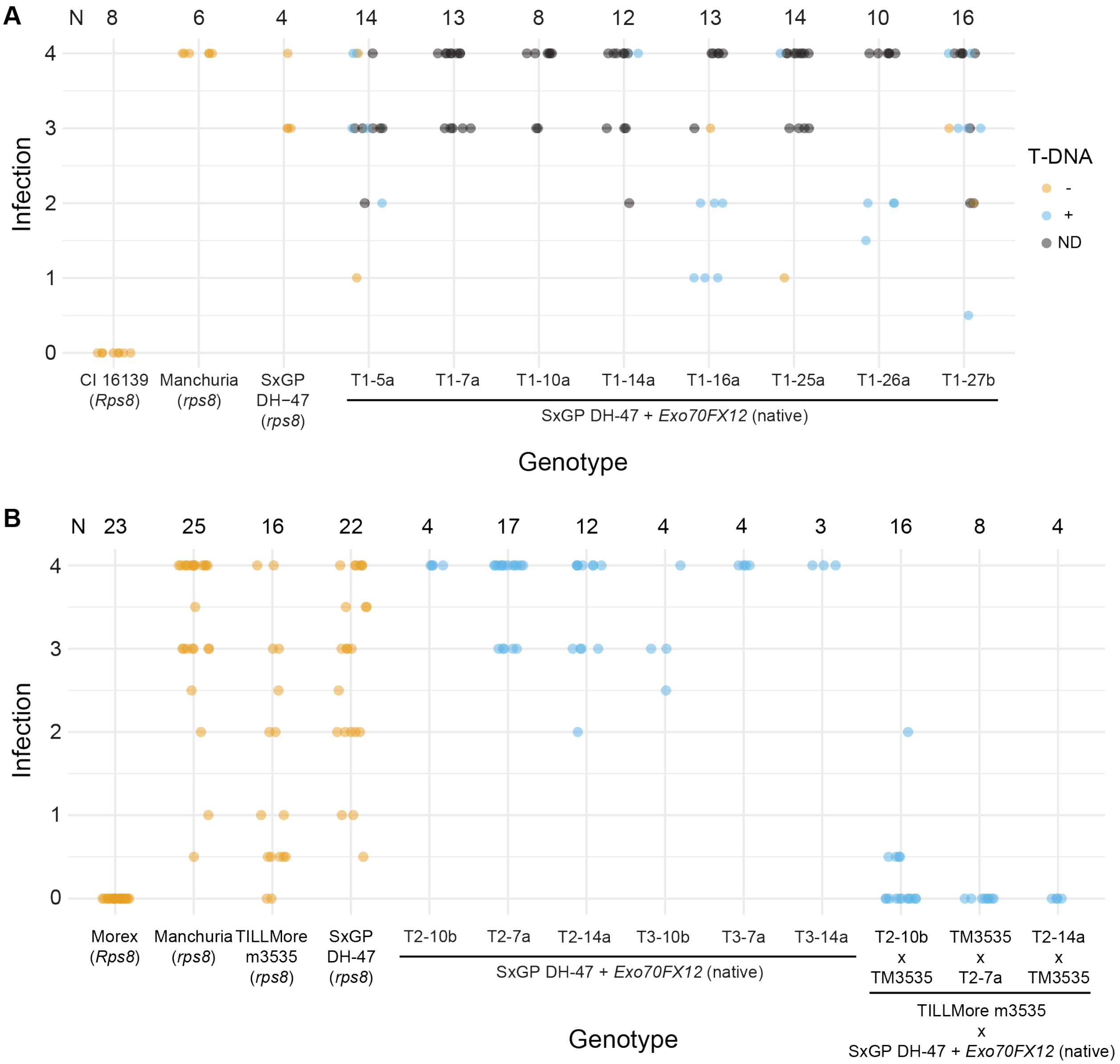

Fig. S10. Transgenic lines natively expressing *Exo70FX12* complement the *rps8* mutant TM3535. (A) Infection phenotypes for independent single copy transformants of barley cv. SxGP DH-47 expressing *Exo70FX12* under native promoters and terminators. **(B)** Infection phenotypes for three independent single copy advanced T_2 and T_3 homozygous transformants of barley cv. SxGP DH-47 expressing *Exo70FX12* under its native promoter and terminator, F_1 based on a cross of transgenic *Exo70FX12* lines and the TM3535 *Exo70FX12* mutant, and controls Morex (*Rps8*), Manchuria (*rps8*), TM3535 (*rps8-TM3535*), and SxGP DH-47 (transformable, susceptible line [*rps8*]). For both panels, presence (blue) or absence (orange) of the T-DNA was determined using a qPCR-based assay. When not determined (ND), data points are in black. Inoculations were performed using *Pst* isolate 16/035 and scored at 14 days post inoculation, N shows the number of evaluated seedlings, and each panel represents a single experiment.

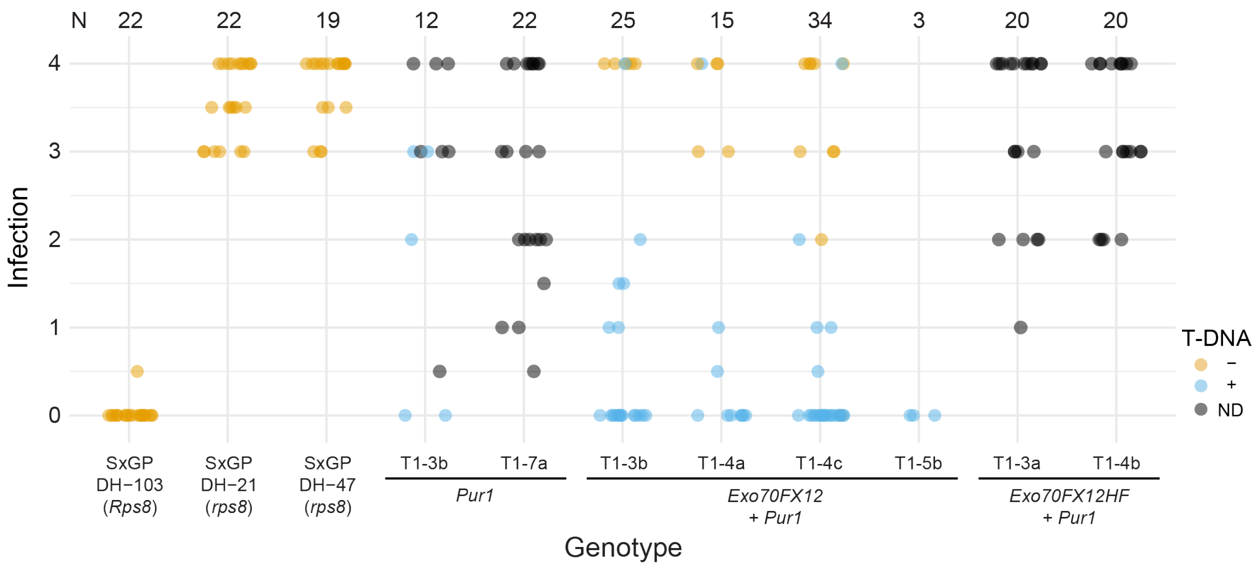

Fig. S11. Extended set of transgenic lines expressing *Pur1, Pur1* and *Exo70FX12*, and *Pur1* with *Exo70FX12* allele from Heils Franken. Infection phenotypes for independent single copy transformants of barley cv. SxGP DH-47 expressing *Pur1*, *Exo70FX12* and *Pur1*, and *Exo70FX12* (Heils Franken allele) and *Pur1*, under their native promoters and terminators. Presence (blue) or absence (orange) of the T-DNA was determined using a qPCR-based assay. When not determined (ND), data points are in black. For both panels, inoculations were performed using *Pst* isolate 16/035 and scored at 14 days post inoculation, N shows the number of evaluated seedlings, and the panel represents a single experiment.

Signal peptide
MMPLVLLFSLLCPSSH

AQPGSPPPPPGPAAEELALLAFKSMLLSDGGSPVLASWNTSSHFCRWSGVACSRQKQ

LRR
VVA LRLGSSN LPGRISPH
LGNLSALTE LDLGGNR LSGEIPPE
LGRLSSLRS LNLSGNF LTGAIPAA
IAAGCTNLTSLVLGPNS LRGTIPAQ
IGTTLRKLTVLDLHRNN LTGHIPPS
LGELRSMQV LSLCFNN LSGEIPAA
LGNLTGLQE LRLHNNG LSGAIPSS
LGLPHDMSY FNLEFNR LTGEIPAS ← Additional LRR domain
IWNLSSLVV FSVMYNM LSGTMPPDA
FAAMPHLHQ IQVNHNQ LHGPFPVS
IANASNISL LQLDSNL FSGIFPRD
IGRLRNLST LLLDNNL FEVHEPRDWGFITE
LTNCSQLRL LGLGGNK FGGVLPDS
LSNLSTSLYDLQLGSNK ITGNIPEG
ISNLINLHN LHMSHNL FTGSLPSS — *rps8-m90* G432R
LGRLQKLAG LYVIGNK LAGPVPST
IGNLTELSD LRLDMNA FSGRIPST
LGKLAKLSS LGLSGNS FIGPIPST
LFSIQKLSMVLDLSHNN FEGSIPQE
IGNLRNLAE LHLESNK FFGEIPTT — *rps8-m2907* A542T
LGECQFLER LYLQNNS LNGSIPST
LNQLKGLQT LDMSSNN LSGQIPKF
LGNMSMLQY MNLSLNR FVGEVPT
FGVFANATA TSVKGNVELCGGIP

Charged residues
TLHLHPCSSQIPKRKHK

Transmembrane
LLVVPIVLSLITTLVVLATLYRLAMG

Charged residues
HKKNRT

Juxtamembrane
ETFSSTSMNSHLKLSYSQLVDATDG

Protein kinase
FSAANFLGSGSFGVVYKGKLQAGHTVAVKVLKLQNPNGFLSFLAECEALRGMRH
RNLVDIITICSSCDTRGYDFKAIVYEFMPNGSLAGWLHPDETEQKQLNLCLRVA — *rps8-m98* Frame shift 1 bp deletion
ILHDVACALHYLHRESPMPIVHCDVKPSNVLLDDDMVAHVGDFGLARGWEFYVE
RNSYLQQSESSMGLTGTTNSSFQQSAESSMGFVRGTIGYAAPEYGAGNMVSPDG — Intron
DIYSYGILVLETVTGKRPTDTIFRHGLRLREYVEQALDHSMMDVIDKRLEDELQ
TADDLSRKKKIDCLMGLLRLGICCSHETPWSRMRTGNIVYML

RTIKESLIERQ

Highly conserved residues in LRR

Invariant residues in protein kinases

19 aa insertion

Nx[ST] N-glycosylation sites

Fig. S12. Domain analysis of Pur1. Domains of Pur1 were annotated based on InterProScan and alignment with Xa21. The Pur1 gene model is HORVU.MOREX.r3.4HG0407750.1. Highly conserved residues in the LRR are shown in bold orange, invariant residues in the protein kinase domain shown in bold green, the site of individual mutations (*rps8-TM90, rps8-TM98, rps8-TM2907*) and their result change to the protein, putative N-glycosylation sites, the position of the single intron, and the 19 amino acid insertion in the protein kinase domain in a pink rectangle. Design of the figure is based on the original characterization of Xa21.

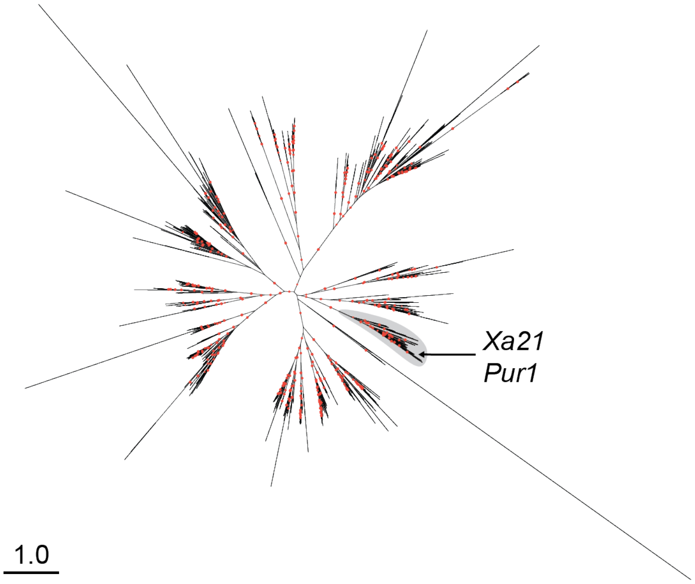

Fig. S13. *Pur1* belongs to the LRR-XII subfamily of RK. Maximum likelihood unrooted phylogenetic tree based on 959 full length RK LRR-XII subfamily proteins from barley (*Hordeum vulgare*), wheat (*Triticum aestivum*), purple false brome (*Brachypodium distachyon*), rice (*Oryza sativa*), carrycillo (*Olyra latifolia*), maize (*Zea mays*), foxtail millet (*Setaria italica*), and sorghum (*Sorghum bicolor*). The *Xa21-Pur1* subclade is defined by the labelled grey region. Red dots indicate bootstrap support greater than 80%. Scale indicates 1.0 substitutions per site.

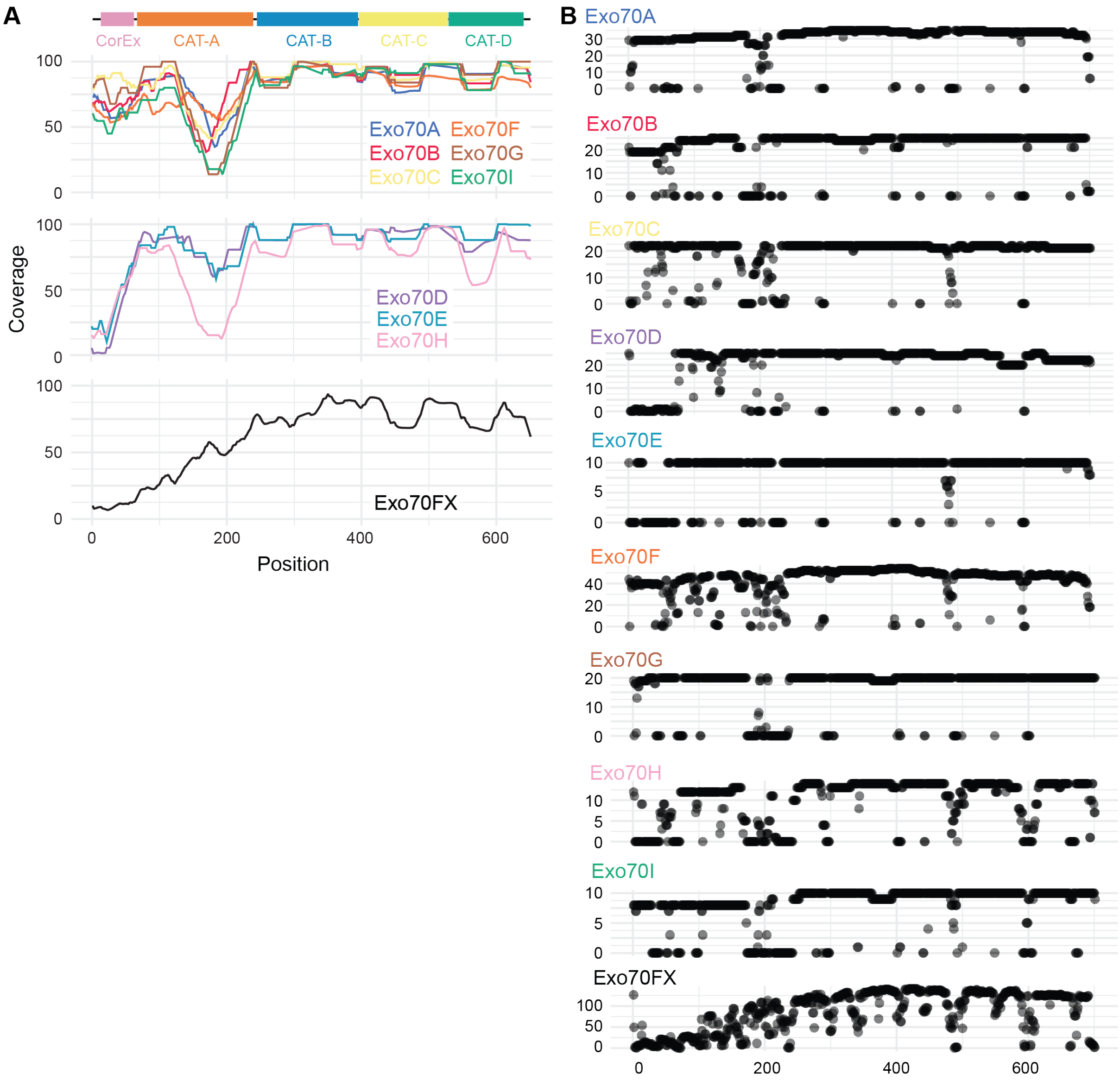

Fig. S14. The Exo70FX family members have a distinct pattern of N-terminal loss as compared to other Exo70 families. (A) Multiple sequence alignment coverage for all grass Exo70 clades. Coverage was estimated for each Exo70 clade using a sliding window of 50 amino acids based on the multiple sequence alignment used to generate the phylogenetic tree shown in Fig. 3a. **(B)** Coverage was computed on every position in the multiple sequence alignment used for the phylogenetic tree shown in **(A)**. The x-axis is the position within the alignment and the y-axis is the coverage based on the total number of family members. Exo70 families Exo70A (N=35), Exo70B (N=25), Exo70C (N=22), Exo70F (N=54), Exo70G (N=20), and Exo70I (N=10) show retention of all five sub-domains, whereas Exo70D (N=25), Exo70E (N=10), and Exo70H (N=14) have lost the CorEx sub-domain. The Exo70FX family (N=143) has a unique coverage pattern with loss of the CorEx and CAT-A sub-domains in the majority of members. Sub-domain structure is based on yeast Exo70.

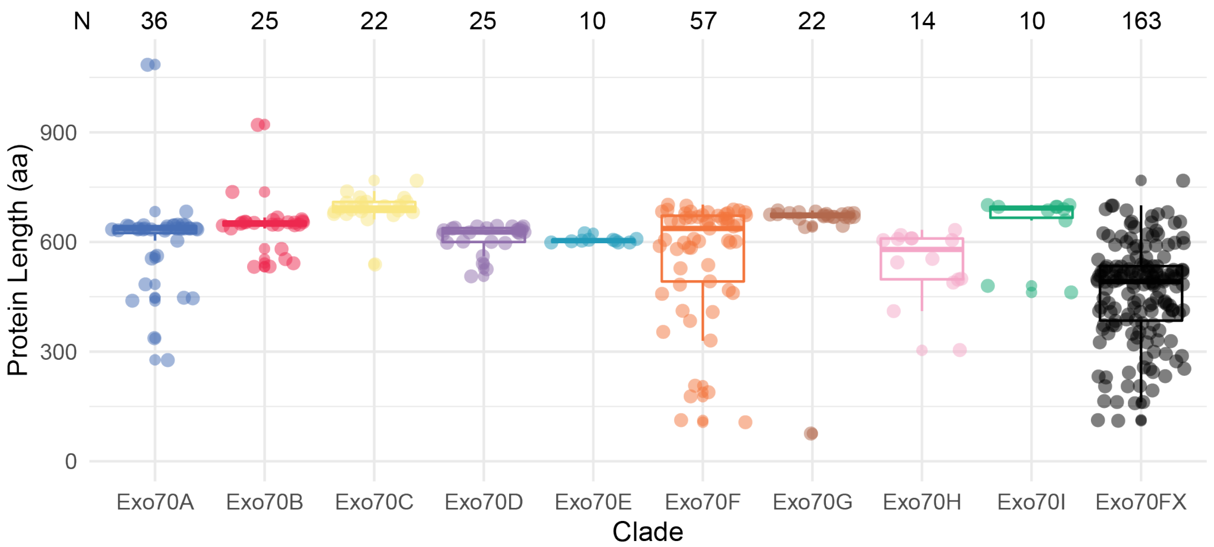

Fig. S15. Members of the Exo70F and Exo70FX clades are experiencing a reduction in protein length. The y-axis is the length of predicted protein sequences (number of amino acids). Exo70 proteins from barley (*H. vulgare*), wheat (*T. aestivum*), purple false brome (*B. distachyon*), rice (*O. sativa*), *O. thomaeum*, maize (*Z. mays*), foxtail millet (*S. italica*), and sorghum (*S. bicolor*). Boxplots showing the 25th, 50th, and 75th quantiles of protein length for Exo70 clade members. Upper and lower whiskers extend to the largest and smallest values no further than 1.5 times the inter-quartile range. Individual lengths are shown using jitter. N denotes the number of proteins in each Exo70 clade.

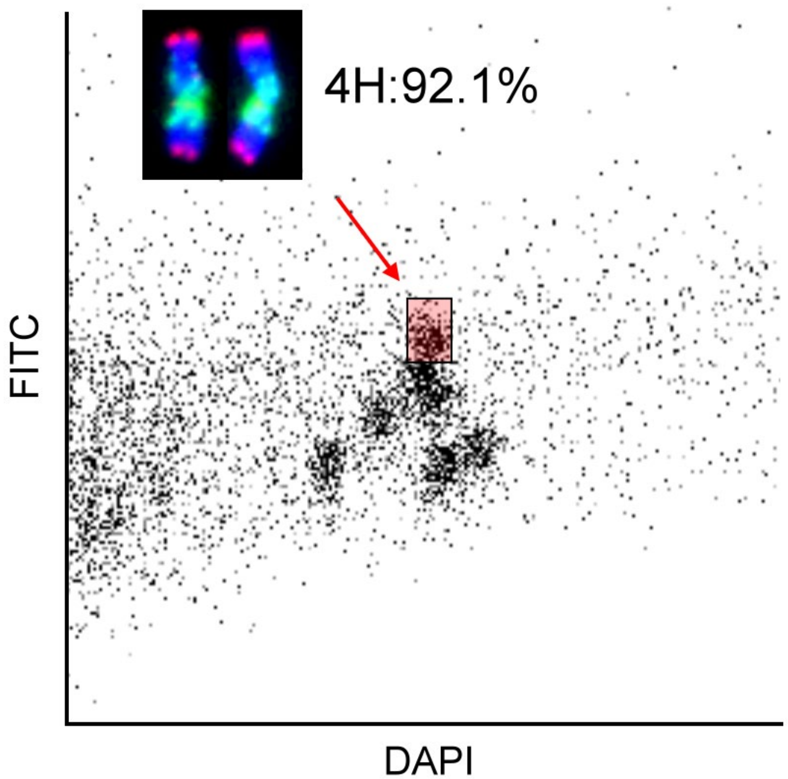

Fig. S16. Bivariate flow karyotype DAPI vs. GAA-FITC obtained after flow cytometric analysis of mitotic metaphase chromosomes isolated from barley accession CI 16139. Barley 4H chromosomes were flow sorted at purity of 92.1% from the sorting window shown as red rectangle. Inset: Images of chromosome 4H flow-sorted onto a microscope slide. The chromosome was identified based on fluorescent labelling after FISH with probes for GAA microsatellite (green) and HvT01 satellite (red). The chromosomes were counterstained by DAPI (blue).

Table S1. Putative and confirmed loss of function mutants in *Rps8*-mediated resistance

Identifier	Infection (Screen)	Infection (Retest)
TM0020	2.00	0.07
TM0090	3.20	2.88
TM0098	3.67	3.21
TM0181	3.75	3.00
TM0343	2.00	1.38
TM0354	1.29	0.00
TM0399	1.33	0.00
TM0758	2.67	0.00
TM0764	3.00	0.00
TM1029	2.00	0.63
TM1048	2.00	0.88
TM1387	3.20	0.31
TM1674	1.29	1.58
TM1781	1.50	1.69
TM2336	1.20	Not tested
TM2357	1.67	0.00
TM2478	1.06	0.13
TM2510	1.00	0.00
TM2512	3.50	0.00
TM2722	3.71	Not tested
TM2810	4.00	Not tested
TM2841	3.30	Not tested
TM2907	4.00	3.94
TM2916	2.25	Not tested
TM2985	3.00	Not tested
TM3013	1.29	2.29
TM3466	3.20	Not tested
TM3535	2.50	3.81
TM3552	2.00	0.44
TM3745	1.20	Not tested
TM4059	3.83	Not tested
TM4087	2.50	0.50
TM4446	1.00	Not tested
TM4590	1.00	Not tested
TM4863	1.00	Not tested
TM5205	2.00	Not tested
TM5392	2.00	Not tested

Table S2. Exo70 families in diverse Poales species

Species	Order	Family	Exo70 family									
			A	B	C	D	E	F	G	H	I	FX
Musa acuminata	Zingiberales	Musaceae	3	3	4	3	2	3	3	6	1	0
Ananas comosus	Poales	Bromeliaceae	3	1	1	2	1	3	1	0	1	0
Streptochaeta angustifolia	Poales	Poaceae	7	8	3	2	3	3	1	4	2	5
Pharus latifolius	Poales	Poaceae	3	2	2	2	1	5	2	2	0	2
Setaria italica	Poales	Poaceae	4	3	2	2	1	5	2	2	1	19
Sorghum bicolor	Poales	Poaceae	3	3	2	2	1	4	2	1	1	16
Oropetium thomaeum	Poales	Poaceae	4	2	2	4	1	7	2	3	1	8
Zea mays	Poales	Poaceae	2	4	4	5	1	7	4	1	1	7
Oryza sativa	Poales	Poaceae	3	3	2	2	1	7	2	2	1	20
Brachypodium distachyon	Poales	Poaceae	4	2	2	2	1	4	2	1	1	7
Hordeum vulgare	Poales	Poaceae	4	2	2	2	1	5	1	1	1	11
Triticum aestivum A genome	Poales	Poaceae	4	2	2	2	1	5	2	1	1	31
Triticum aestivum B genome	Poales	Poaceae	4	2	2	2	1	5	2	1	1	33
Triticum aestivum D genome	Poales	Poaceae	4	2	2	2	1	7	2	1	1	11

Table S3. Primers used for molecular cloning

Method	Primer	Sequence (5'-3')
PCR	SH_12_p1f	GGAAGGGAATAACCAACTAG
PCR	SH_12_p1r	CCATCTGTGGCAATCAAGGA
Gibson	Exo70FX12-pBract-p1f	AGAGGTTTCTTGGGTTGAAAGATCCACTAGTTCTAGAGCG
Gibson	Exo70FX12-pBract-p1r	CGCTCTAGAACTAGTGGATCTTTCAACCCAAGAAACCTCT
Gibson	pBract-Exo70-p1f	TAAGCTTGATATCGAATTCCCTACGAAACTGAATATTTAG
Gibson	pBract-Exo70-p1r	CTAAATATTCAGTTTCGTAGGGAATTCGATATCAAGCTTA

Table S4. Pfam identifiers for kinase domains.

Name	Pfam
Pkinase	PF00069
PK_Tyr_Ser-Thr	PF07714
APH	PF01636
PI3_PI4_kinase	PF00454
ABC1	PF03109
PIP5K	PF01504
Pkinase_fungal	PF17667
RIO1	PF01163
Choline_kinase	PF01633
EcKinase	PF02958
HipA_C	PF07804
IPK	PF03770
Fructosamin_kin	PF03881
Kdo	PF06293
CotH	PF08757
IucA_IucC	PF04183
Alpha_kinase	PF02816
DUF2252	PF10009
PIP49_C	PF12260
Ins_P5_2-kin	PF06090
APH_6_hur	PF04655
Pan3_PK	PF18101
Haspin_kinase	PF12330
KIND	PF16474
Fam20C	PF06702
AceK	PF06315
DUF1679	PF07914
DUF4135	PF13575
FTA2	PF13095
Act-Frag_cataly	PF09192
YrbL-PhoP_reg	PF10707
YukC	PF10140
WaaY	PF06176
Kinase-like	PF14531
Kinase-PolyVal	PF18762
Pox_ser-thr_kin	PF05445
UL97	PF06734
Seadorna_VP7	PF07387

Movie S1. (separate file)
Structural model of Pur1 LRR ectodomain based on AlphaFold. The additional LRR repeat is shown in light blue, the amino acids G432 and A542 in green and dark blue, respectively.

Movie S2. (separate file)
Structural model of Exo70FX12 based on AlphaFold. Sub-domains of Exo70 include CAT-A (light blue), CAT-B (green), CAT-C (yellow), and CAT-D (dark blue). Amino acids L130 and E271 in orange and pink, respectively.

Data S1. (separate file)
Protein encoding genes in the *Rps8* region.

Data S2. (separate file)
Association of barley diversity and *Rps8*-mediated resistance.

Data S3. (separate file)
Genomes of monocot species used for gene family analysis.

Data S4. (separate file)
Presence/absence variation of Pur1 and Exo70FX12 in diverse grass species.

Data S5. (separate file)
LRR-RK family XII receptor kinases from diverse grass species and their relationship to *Xa21*.

Data S6. (separate file)
Annotation of Exo70 families in eight Poaceae species.

Data S7. (separate file)
Exo70FX family members in the Exo70FX10, Exo70FX11, Exo70FX12, and Exo70FX15 clades in diverse grass species.

Data S8. (separate file)
Genetic markers used to map *Rps8*.
